# TAZ Expression as a Prognostic Indicator in Colorectal Cancer

**DOI:** 10.1371/journal.pone.0054211

**Published:** 2013-01-23

**Authors:** Hiu-Fung Yuen, Cian M. McCrudden, Yu-Han Huang, Jill M. Tham, Xiaoqian Zhang, Qi Zeng, Shu-Dong Zhang, WanJin Hong

**Affiliations:** 1 Institute of Molecular and Cell Biology, Agency for Science, Technology and Research (A*STAR), Singapore, Singapore; 2 Centre for Cancer Research and Cell Biology (CCRCB), Queen's University Belfast, Belfast, United Kingdom; 3 Department of Biochemistry, Yong Loo Lin School of Medicine, National University of Singapore, Singapore, Singapore; 4 School of Pharmaceutical Sciences, Xiamen University, Xiamen, Fujian, People's Republic of China; University of Newcastle, United Kingdom

## Abstract

The Hippo pathway restricts the activity of transcriptional coactivators TAZ (*WWTR1*) and YAP. TAZ and YAP are reported to be overexpressed in various cancers, however, their prognostic significance in colorectal cancers remains unstudied. The expression levels of TAZ and YAP, and their downstream transcriptional targets, *AXL* and *CTGF*, were extracted from two independent colon cancer patient datasets available in the Gene Expression Omnibus database, totaling 522 patients. We found that mRNA expressions of both TAZ and YAP were positively correlated with those of *AXL* and *CTGF* (*p*<0.05). High level mRNA expression of TAZ, *AXL* or *CTGF* significantly correlated with shorter survival. Importantly, patients co-overexpressing all 3 genes had a significantly shorter survival time, and combinatorial expression of these 3 genes was an independent predictor for survival. The downstream target genes for TAZ-AXL-CTGF overexpression were identified by Java application MyStats. Interestingly, genes that are associated with colon cancer progression (*ANTXR1*, *EFEMP2*, *SULF1*, *TAGLN*, *VCAN*, *ZEB1* and *ZEB2*) were upregulated in patients co-overexpressing TAZ-AXL-CTGF. This TAZ-AXL-CTGF gene expression signature (GES) was then applied to Connectivity Map to identify small molecules that could potentially be utilized to reverse this GES. Of the top 20 small molecules identified by connectivity map, amiloride (a potassium sparing diuretic,) and tretinoin (all-trans retinoic acid) have shown therapeutic promise in inhibition of colon cancer cell growth. Using MyStats, we found that low level expression of either *ANO1* or *SQLE* were associated with a better prognosis in patients who co-overexpressed TAZ-AXL-CTGF, and that *ANO1* was an independent predictor of survival together with TAZ-AXL-CTGF. Finally, we confirmed that TAZ regulates Axl, and plays an important role in clonogenicity and non-adherent growth *in vitro* and tumor formation *in vivo*. These data suggest that TAZ could be a therapeutic target for the treatment of colon cancer.

## Introduction

The Hippo pathway plays an important role in cell proliferation, organ size control, and cancer development and progression [1], [2], [3], [4]. YAP and TAZ are both transcriptional co-activators that are inhibited by the Hippo pathway [1], [2], [3], [4]. Aberrant inactivation of the Hippo pathway and/or overexpression of TAZ and YAP results in transcriptional activation of their downstream targets [1], [2], [3], [4].

YAP overexpression induces cell proliferation and epithelial mesenchymal transition (EMT), and inhibits apoptosis and contact inhibition [5], [6], [7]. Transcriptional activation of epidermal growth factor receptor ligand amphiregulin may account for YAP-mediated induction of cell proliferation, especially under serum-depletion [7], while YAP also cooperates with Myc to promote cell proliferation [8]. Recently, YAP has been shown to play a critical role in stem cell biology. It is induced during pluripotent stem cell reprogramming, whilst silencing of YAP reduces the pluripotency of embryonic stem cells [9]. YAP promotes ovarian cancer progression, and high levels of nuclear expression are inversely associated with patient survival [10]. In particular, YAP is associated with clear cell ovarian tumors, an ovarian malignancy subtype with poor prognosis [11]. YAP has also been shown to play an oncogenic role in esophageal squamous cell carcinoma [12]. In liver cancer, microRNA-mediated inhibition of YAP inhibits tumor characteristics including cell proliferation and invasion [13]. Conversely, there are reports showing an opposite, tumor suppressive, role of YAP in promoting p73-mediated apoptosis [14], [15]. In breast and head and neck cancers, YAP has been shown to act as a tumor suppressor in certain circumstances [14], [16].

TAZ is structurally homologous to YAP, is likewise inhibited by the Hippo pathway, and also promotes EMT-mediated cancer progression [17], [18], [19]. TAZ regulates mesenchymal stem cell differentiation by modulating Runx2- and PPARgamma-dependent gene expression [20], as well as stem cell self-renewal through controlling localization of Smad [21]. TAZ plays an important role in the progression of breast [22], [23] and non-small cell lung cancer [24], [25]. Importantly, TAZ confers cancer stem cell-related traits on breast cancer cells, further highlighting its importance in tumor initiation and progression [26]. TAZ is also overexpressed in papillary thyroid carcinoma [27].

TAZ and YAP have been shown to interact with several transcriptional factors [2], [3], [4], with the TEAD family of transcriptional factors (TEAD1-4) being the most relevant in cell proliferation and cancer progression [19], [28], [29]. The X-ray crystal structures of YAP-TEAD complexes have been resolved and the proposed interaction is supported by and consistent with functional analysis [30], [31], [32], showing that YAP-TEAD complexes activates gene transcription.

YAP expression was observed in colon adenocarcinoma [33], [34], [35]. It is overexpressed in human colon cancer specimens and overexpression of YAP promotes cell proliferation and survival in colon cancer cells [36]. Recent findings show that knockdown of TAZ results in a decrease in cell proliferation in culture and tumor growth *in vivo* [26].

Despite evidence suggesting the potential implication of YAP and TAZ in colon cancer progression, their prognostic significance in colorectal cancer is unknown. In this study, we analyzed the mRNA expression of YAP and TAZ, and two of its downstream target genes, *AXL* and *CTGF*, in two independent colon cancer patient cohorts comprising 522 patients. We found that TAZ, but not YAP, is a prognostic marker in colon cancer progression. Furthermore, TAZ-*AXL-CTGF* co-overexpression, which defines both the expression of TAZ and its transcriptional activity on target gene expression, is a novel prognostic indicator, that is independent of tumor grade and stage, for colon cancer patients. The role of TAZ in colon cancer cell proliferation and oncogenesis was validated by functional study.

## Materials and Methods

### Extraction of clinical and microarray gene expression data from colon cancer patient datasets

Two colon cancer patient datasets, GSE14333 [37] and GSE17538 [38], available in the Gene Expression Omnibus (GEO) Database (http://www.ncbi.nlm.nih.gov/gds) were included in this study. The GEO website has standardized URLs for its individual datasets, e.g. the overall summary information about the microarray dataset GSE14333 can be accessed at http://www.ncbi.nlm.gov/geo/query/acc.cgi?acc=GSE14333. For each GEO data series, links are provided at the bottom of the page to the Series Matrix File(s), which contain the expression values for each gene (probeset) and each microarray. The URLs to the Series Matrix File(s) are also standardized. For GSE14333, the URL was ftp://ftp.ncbi.nlm.nih.gov/pub/geo/DATA/SeriesMatrix/GSE14333. The files in gzip format were then unzipped to the tab-delimited text format, which contain detailed information for statistical analysis. The GSE14333 and GSE17538 datasets are the two largest colon cancer patient datasets on the database, and comprise 522 patients, 458 of whose survival data is available in the database. The GSE17538 data series consists of four SubSeries: GSE17536, GSE17537, GSE19072 and GSE19073. GSE19072 and GSE19073 were excluded from this study as they lack clinical data. Microarray gene expression data were retrieved from the data matrices deposited to the GEO database by the original authors. The gene expression levels in GSE14333 and GSE17538 are represented by base-2 logarithm of the MAS5 value and the RMA values, respectively, as adopted by the original authors. R scripting was used to extract the expression values of a small number of genes (probesets) of interest and the clinical data from the data matrixes downloaded from GEO.

### Demographic and clinical data of the two patient cohorts

Both age and gender of the patients were available demographic data in the two datasets analyzed in the present study. Patients had a median age of 67 years (Range: 26–92 year-old) and 65.5 years (Range: 23–94 year-old) in GSE14333 and GSE17538, respectively. There were 43% and 46% female, respectively, in cohort GSE14333 and GSE17538. The GSE14333 cohort consists of 290 patients, for whom 226 survival data were available. Fifteen, 32, 31 and 21% of patients had stage A, B, C and D tumors, respectively. The survival data were not available for all the patients of stage D. Survival data were available for all 232 patients in the GSE17538 cohort. Twelve, 31, 33 and 24% patients had tumors of AJCC stage I, II, III and IV, respectively, and 8, 78 and 14% of patients had Grade A, B and C tumors respectively.

### Correlations of gene expression levels and clinical data

All statistical analyses were performed using SPSS19.0. The associations between expression levels of genes were analyzed by Spearman's rank test. Expression levels were further divided into high and low levels using median expression level as the cut-off point for Kaplan-Meier survival analysis. Results were compared by log-rank test. Univeriate Cox regression analysis was used to correlate the gene expression levels and patient survival and multivariate Cox regression analysis was used to identify independent predictors for patient survival using a backward stepwise approach with an entry limit of p<0.1 and a removal limit of p>0.05. Patients were divided further into 4 groups based on the expression levels of TAZ, AXL and CTGF; the TAZ-AXL-CTGF-low group consisted of patients who expressed all these 3 genes at low levels; the TAZ-AXL-CTGF-intermediate-low group consisted of patients who expressed one of these three genes at a high level; the TAZ-AXL-CTGF-intermediate-high group consisted of patients who expressed two of these three genes at high levels; the TAZ-AXL-CTGF-high group consisted of patients who expressed all three genes at high levels. The survival time of patients stratified by this grouping method were analyzed by Kaplan-Meier analysis and Cox regression as described above.

### Identification of TAZ-AXL-CTGF co-expressing genes

Patients were stratified into four groups based on the expression levels of TAZ, AXL and CTGF as described above. The gene expression patterns of patients in TAZ-AXL-CTGF low subgroup and those in the TAZ-AXL-CTGF high subgroup (whose survival was significantly poorer) were compared. Probesets that were differentially expressed between these two subgroups were identified by 2-sample Welch's T-test. This test was used to avoid the type I error due to unequal variances of the values of probesets between subgroups. Briefly, a Welch's t test was applied to each probeset corresponding to a certain gene in the data matrix using our own Java application MyStats. P values and the differential expression in fold changes for all the probesets were generated as tab-delimited worksheets of Excel for further analysis. The genes were prioritized by ascending p-values. The top 100 probesets were prioritized in both patient datasets, and the genes common to both datasets were analyzed further.

### Identification of potential inhibitory compounds targeting TAZ-AXL-CTGF overexpressing colon cancer (Connectivity Map)

Gene expression connectivity mapping was performed using Statistically Significant Connection's Map (sscMap) to identify candidate small molecule compounds that may inhibit the expression of genes that are co-regulated in TAZ-AXL-CTGF co-expressing aggressive colon cancer [39], [40], [41]. Of the probesets identified to be co-expressed with TAZ-AXL-CTGF, 33 were present on the Affymetrix HG-U133A microarray platform, which was used to generate the microarray database for the Connectivity Map [39]. The compiled gene signature was then fed to the Java application sscMap [41] as a query signature, and its association with the 6000 gene expression profiles generated by treating cancer cells with over 1000 small molecules were compared. The gene signature perturbation procedure, which increases the specificity of the output results, was applied as previously described [42]. All the small molecular compounds, that were negatively associated with the TAZ-AXL-CTGF-GES, were sorted and ranked by their *p*-value, perturbation stability and standardized connection score. The *p*-value that was considered significant was set at a stringent threshold (p = 1/1309), ensuring that the results generated by sccMap yield only maximally one expected false positive small molecule over the 1309 small molecules tested in the sccMap [42]. The top 20 small molecules were then entered into the Pubmed (www.pubmed.com) search engine together with colon cancer to identify research articles that have described their effects of the particular molecules on treatment of colon cancer.

### Identification of therapeutic targets for colon cancer patients overexpressing TAZ-AXL-CTGF

Patients who co-overexpressed TAZ-AXL-CTGF were stratified into two groups based on their survival statuses. Differential expressions of different probesets between patients in the TAZ-AXL-CTGF-alive subgroup and those in the TAZ-AXL-CTGF-deceased subgroup were identified as described above.

### Cell culture and retroviral transduction

HCT116 and SW620 cells were obtained from American Type Culture Collection and maintained in F12K/DME medium supplemented with 10% Fetal Bovine Serum (FBS), 10 ug/ml Penicillin/Streptomycin (P/S) (Life technologies, Carlsbad, CA). The amphotropic Phoenix packaging cell line was obtained from the Nolan Laboratory (Stanford University) and maintained in DMEM medium supplemented with 10% FBS and 10 ug/ml P/S. Retroviral infection was performed as previously described [18]. The short hairpin (shRNA) against human TAZ construct (5′-AGGTACTTCCTCAATCACA-3′) carried by the pSuperRetro-puro vector (shTAZ) was used for TAZ knockdown (Oligoengine, Seattle, WA) while the scramble shRNA construct (5′-CCTAAGGTTAAGTCGCCCTCG-3′; shScr) was used for control. Stable cell lines were established by selecting the traduced cells in 2 ug/ml puromycin (Sigma Aldrich, St. Louis, MO).

### Western blot analysis

Immunoblotting was performed as previously described [18] using SuperSignal West Pico (Thermo Scientific, Rockford, IL). The commercial TAZ antibody was obtained from Imgenex (San Diego, CA).

### Anchorage-independent soft agar and clonogenic assays

For the soft agar assay, 5000 cells resuspended in 0.35% (w/v) agarose in culture medium were overlaid on a solidified 0.5% (w/v) agarose in culture medium. The upper layer was allowed to solidify. Medium with puromycin was added the following day and cells were then incubated at 37°^-^C with 5% CO_2_. Fresh medium with puromycin was supplemented and the colonies formed were stained with 1 mg/ml Thiazolyl Blue Tetrazolium Bromide (Sigma Aldrich) for 4 hours in the incubator at 37°C with 5% CO_2_. The excess dye was removed by destaining multiple times with water and the number of colonies was determined.

For clonogenic assay, 500 cells were seeded per well in triplicate in 6-well plate. The culture medium was refreshed every week and the cells were fixed with 4% paraformaldehyde in PBS after 2 weeks incubation at 37°C with 5% CO_2_. The fixed cells were stained with 0.5% crystal violet (Sigma Aldrich) in 20% ethanol overnight. The cells were rinsed with water, dried and colony number was analyzed.

### Tumorigenesis in nude mice

Hundred ul of a cell suspension of 1.5×10^7^/ml were inoculated subcutaneously in the left and right hind flanks of four-to-six week-old female nude mice. Tumor development was monitored sfter 2 weeks. Mice were then euthanized and the tumors were removed for analysis.

## Results

### TAZ and YAP mRNA expressions positively correlate with mRNA expression of their downstream target genes, AXL and CTGF

**Figure 1:**
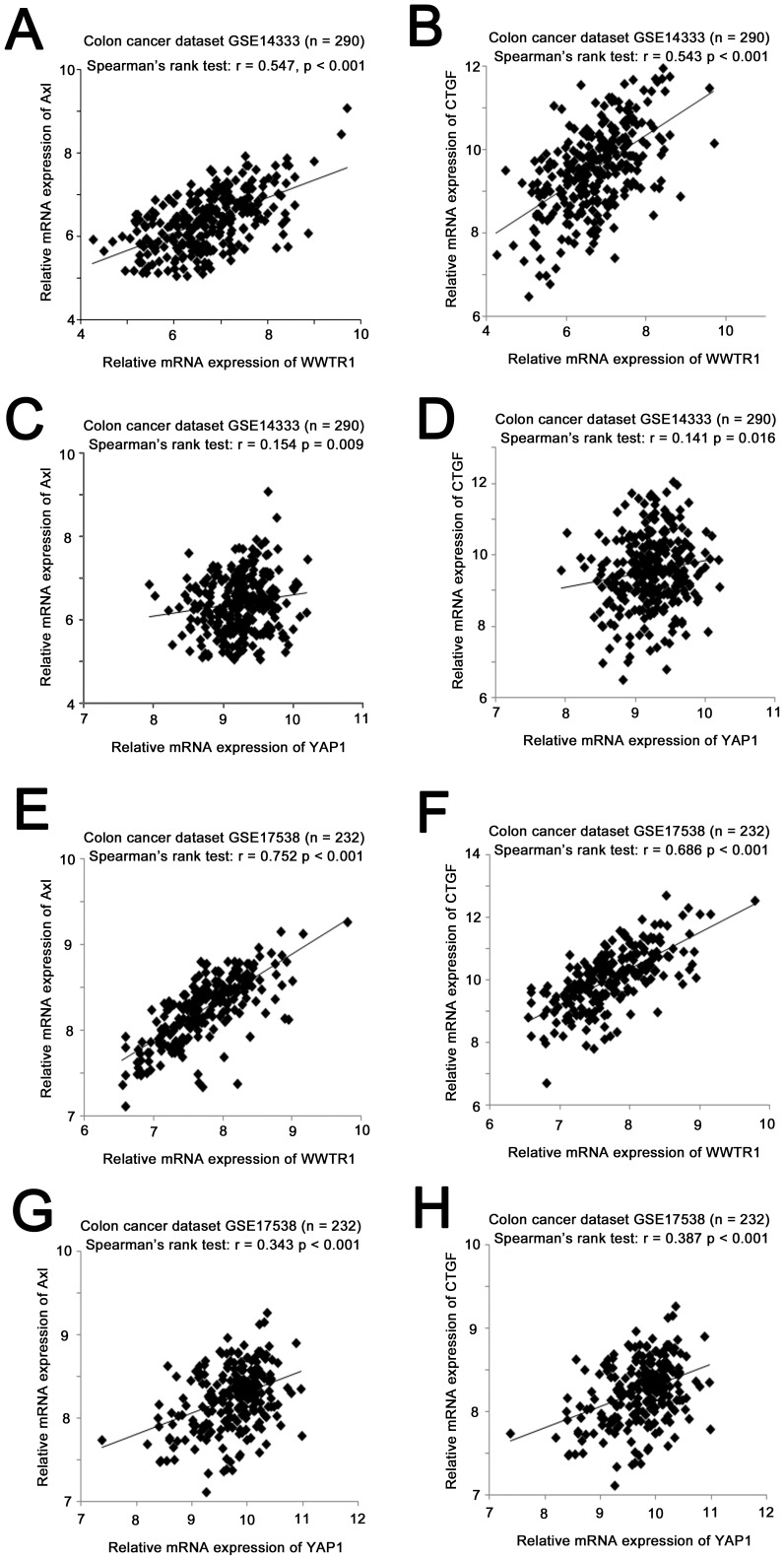
The correlations among mRNA expression of TAZ (WWTR1), YAP (YAP1), AXL and CTGF in colon cancer specimens. Scatter plots for (A) TAZ mRNA expression versus *AXL* mRNA expression, (B) TAZ mRNA expression versus *CTGF* mRNA expression, (C) YAP mRNA expression versus *AXL* mRNA expression, and (D) YAP mRNA expression versus *CTGF* mRNA expression in the GSE14333 colon cancer datasets, and (E) TAZ mRNA expression versus *AXL* mRNA expression, (F) TAZ mRNA expression versus *CTGF* mRNA expression, (G) YAP mRNA expression versus *AXL* mRNA expression, and (H) YAP mRNA expression versus *CTGF* mRNA expression in the GSE17538 colon cancer datasets.

Previously, we and others have shown that *AXL* and *CTGF* are two important downstream target genes of TAZ and YAP [23], [43], [44]. In the present study, we investigated whether the mRNA expression levels of the two transcriptional co-activators in the Hippo pathway, TAZ and YAP, correlate with the mRNA expression of *AXL* and *CTGF*. In the 290 colon cancer patients from the GSE14333 dataset, TAZ expression was significantly correlated with both AXL (Spearman's rank test, *r* = 0.547, *p*<0.001; Figure 1A) and CTGF (*r* = 0.543, *p*<0.001; Figure 1B) expressions. YAP mRNA expression was also positively correlated with AXL (*r* = 0.154, *p* = 0.009; Figure 1C) and CTGF (*r* = 0.141, *p* = 0.016; Figure 1D) mRNA expression in the same dataset, but to a lesser extent. In 232 colon cancer patients from GSE17538, TAZ mRNA expression was significantly positively correlated with both AXL (*r* = 0.752, *p*<0.001; Figure 1E) and CTGF (*r* = 0.686, *p*<0.001; Figure 1F) mRNA expressions, while YAP mRNA was also significantly positively correlated with mRNA expression of both genes, again to a lesser extent (AXL: *r* = 0.343, *p*<0.001; Figure 1G and CTGF: *r* = 0.387, *p*<0.001; Figure 1H).

### TAZ, but not YAP, mRNA expression is a predictor for patient survival

**Figure 2:**
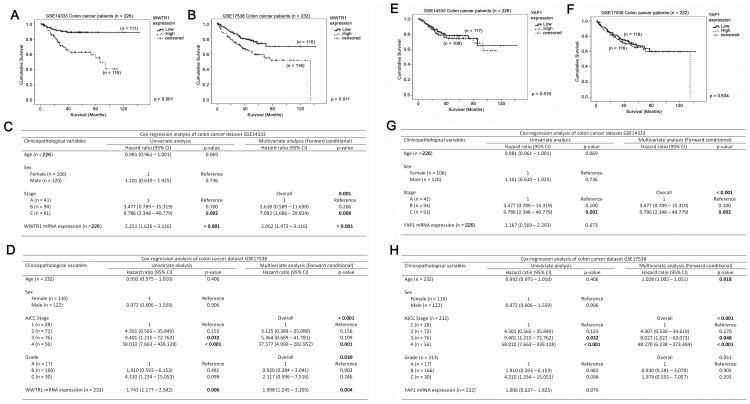
The associations between TAZ or YAP, and survival in colon cancer patients. Kaplan-Meier analyses for TAZ mRNA expression in (A) GSE14333 and (B) GSE17538 colon cancer patient datasets. Univariate and Multivariate Cox regression analyses for TAZ mRNA expression, age, tumor stage and tumor grade in (C) GSE14333 and (D) GSE17538 colon cancer patient datasets. Kaplan-Meier analyses for YAP mRNA expression in (E) GSE14333 and (F) GSE17538 colon cancer patient datasets. Univariate and Multivariate Cox regression analyses for TAZ mRNA expression, age, tumor stage and tumor grade in (G) GSE14333 and (H) GSE17538 colon cancer patient datasets.

**Figure 3:**
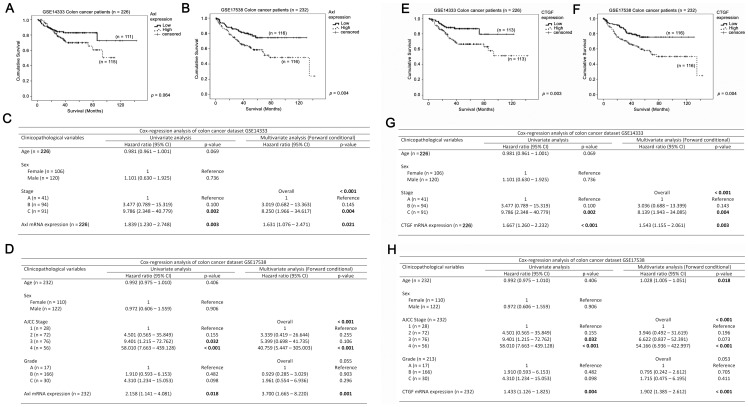
The associations between AXL or CTGF, and survival in colon cancer patients. Kaplan-Meier analyses for *AXL* mRNA expression in (A) GSE14333 and (B) GSE17538 colon cancer patient datasets. Univariate and Multivariate Cox regression analyses for *AXL* mRNA expression, age, tumor stage and tumor grade in (C) GSE14333 and (D) GSE17538 colon cancer patient datasets. Kaplan-Meier analyses for *CTGF* mRNA expression in (E) GSE14333 and (F) GSE17538 colon cancer patient datasets. Univariate and Multivariate Cox regression analyses for *CTGF* mRNA expression, age, tumor stage and tumor grade in (G) GSE14333 and (H) GSE17538 colon cancer patient datasets.

In the 226 patients whose survival data were available from the GSE14333 colon cancer patient cohort, a high level of TAZ mRNA expression was significantly correlated with a shorter survival (high level: mean survival = 72.3 months, 95% Confidence Interval (CI) = 63–81 months; low level: mean survival = 129 months, 95% CI = 121–136 months, *p*<0.001; Figure 2A). By Cox-regression analysis, TAZ mRNA expression was significantly correlated with survival (Hazards Ratio (HR) = 2.251, 95% CI = 1.626–3.116, *p*<0.001; Figure 2C) in the GSE14333 colon cancer patient cohort. By multivariate analysis (Figure 2C), TAZ mRNA expression (HR = 2.062, 95% CI = 1.472–3.116, *p*<0.001) and tumor staging (*p*<0.001) are both independent predictors of survival in the same cohort. Similarly, a high level of TAZ mRNA expression was significantly correlated with shorter patient survival in the GSE17538 colon cancer patient cohort (high level: mean survival = 84 months, 95% CI = 72–96 months; low level: mean survival = 109 months, 95% CI = 97–120 months, *p* = 0.011; Figure 2B). By Cox-regression, TAZ mRNA expression is a predictor of survival in this cohort (HR = 1.743, 95% CI = 1.177–2.582, *p* = 0.006; Figure 2D). Mulivariate Cox-regression analysis also showed that TAZ mRNA expression (HR = 1.998, 95% CI = 1.245–3.205, *p* = 0.004; Figure 2D) was an independent predictor for survival together with stage (*p*<0.001) and grade (*p* = 0.03) of the cancers in this colon cancer patient cohort. On the other hand, YAP mRNA expression did not significantly correlate with patient survival by Kaplan-Meier analysis (GSE14333: *p* = 0.519; Figure 2E and GSE17538: *p* = 0.634; Figure 2F) or by Cox-regression analysis (GSE14333: *p* = 0.673; Figure 2G and GSE17538: *p* = 0.979; Figure 3H) in either dataset. These results suggest that TAZ mRNA expression is a novel prognostic marker for colon cancer patients, but YAP is not.

### Both AXL and CTGF, downstream target genes of TAZ and YAP, are predictors of patient survival

To investigate whether the functional outcome of the transcriptional program mediated by TAZ and YAP confers prognostic value in colon cancer patients, we analyzed whether the mRNA expression of AXL and CTGF, two of the most well defined target genes of TAZ/YAP-TEAD complexes, correlate with survival of patients in the two colon cancer patient cohorts.

High level mRNA expression of AXL correlated with a shorter colon cancer patient survival time in the GSE14333 cohort, although this correlation was not significant (high level: mean survival = 80 months, 95% CI = 71–88 months; low level: mean survival = 114 months, 95% CI = 101–129 months, *p* = 0.064; Figure 3A). In the GSE17538 cohort, high level mRNA expression of AXL was significantly correlated with shorter survival (high level: mean survival = 84 months, 95% CI = 72–96 months; low level: mean survival = 104 months, 95% CI = 94–114 months, *p* = 0.004; Figure 3B). By Cox-regression analysis, AXL mRNA expression was a predictor of patient survival in both the GSE14333 (HR = 1.839, 95% CI = 1.230–2.748, *p* = 0.003; Figure 3C) and the GSE17538 (HR = 2.158, 95% CI = 1.141–4.081, *p* = 0.018; Figure 3D) cohorts. In the GSE14333 cohort, AXL mRNA expression (HR = 1.631, 95% CI = 1.076–2.471, *p* = 0.021; Figure 3C) was an independent predictor of patient survival with tumor staging (*p*<0.001), while in the GSE17538 cohort, AXL mRNA expression (HR = 3.700, 95% CI = 1.665–8.220, *p* = 0.001; Figure 3D) was an independent predictor of patient survival together with stage (*p*<0.001) and grade (*p* = 0.055) of the cancers.

Similar results were obtained for CTGF. A high level of CTGF mRNA expression was significantly correlated with shorter patient survival in both GSE14333 (high level: mean survival = 87 months, 95% CI = 74–102 months; low level: mean survival = 98 months, 95% CI = 88–108 months, *p* = 0.012; Figure 3E) and GSE17538 (high level: mean survival = 85 months, 95% CI = 73–97 months; low level: mean survival = 105 months, 95% CI = 95–114 months, *p* = 0.004; Figure 3F) cohorts. Univariate Cox-regression analysis also showed that CTGF mRNA expression was a predictor for patient survival in both cohorts (GSE14333: HR = 1.667, 95% CI = 1.260–2.232, *p*<0.001; Figure 3G and GSE17538: HR = 1.433, 95% CI = 1.126–1.825, *p* = 0.004; Figure 3H). Moreover, CTGF mRNA expression is an independent predictor for survival in both cohorts (GSE14333: HR = 1.543, 95% CI = 1.155–2.061, *p*<0.001; Figure 3G and GSE17538: HR = 1.902, 95% CI = 1.385–2.612, *p*<0.001; Figure 3H) together with stage (GSE14333: *p*<0.001 and GSE17538: *p*<0.001) and grade (GSE17538: *p* = 0.053) of the cancers.

### TAZ, AXL and CTGF can be used in combination to predict colon cancer patient survival

**Figure 4:**
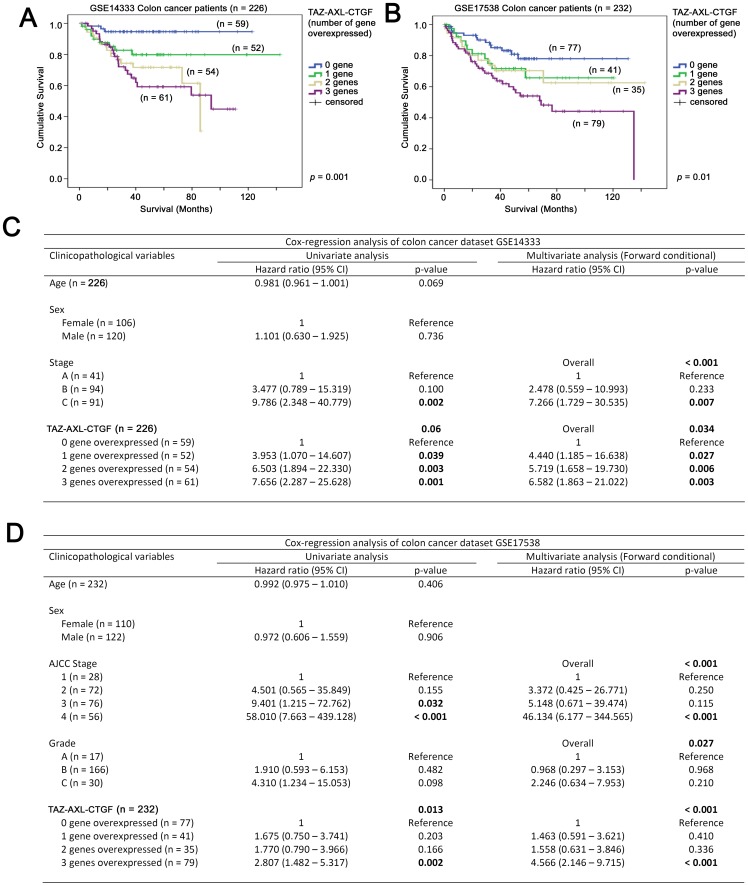
The associations between TAZ-AXL-CTGF expression and survival in colon cancer patients. Patients were divided into 4 groups according to the number of genes that they overexpressed (expressed at above the Median level) among TAZ, *AXL* and *CTGF*. Kaplan-Meier analyses for TAZ-AXL-CTGF mRNA expression in (A) GSE14333 and (B) GSE17538 colon cancer patient datasets. Univariate and Multivariate Cox regression analyses for TAZ-AXL-CTGF mRNA expression, age, tumor stage and tumor grade in (C) GSE14333 and (D) GSE17538 colon cancer patient datasets.

As described above, expression of TAZ, *AXL* and *CTGF* all correlated with colon cancer patient survival. We further investigated whether their expression could be used in combination as a prognostic marker for colon cancer patients. In the GSE14333 cohort, patients whose tumors had low level expression of the three genes had a mean survival time of 117 months (95% CI = 110–123 months), while those whose tumors overexpressed only one of the three genes also had a mean survival of 117 months (95% CI = 101–132 months). Patients whose tumors had a high level expression of two of the three genes had a mean survival time of 65 months (95% CI = 57–74 months), while those whose tumors had a high level expression of all three genes had a mean survival time of 72 months (95% CI = 60–84 months). The survival time of the patients between these four subgroups were significantly different (*p* = 0.001; Figure 4A). In Cox-regression analysis, using the subgroup containing patients whose tumors had a low expression of all three genes as a reference group, we found that patients whose tumors overexpressed one (HR = 3.953, 95% CI = 1.070–14.607, *p* = 0.039), two (HR = 6.503, 95% CI = 1.894–22.330, *p* = 0.003) or all the three genes (HR = 7.656, 95% CI = 2.287–25.628, *p* = 0.001) had an increasing risk for disease progression (Figure 4C).

Similar results were obtained in the GSE17538 patient cohort. Patients whose tumors expressed the three genes at low level had a mean survival time of 108 months (95% CI = 97–119 months), those whose tumors had a high level expression of one of the three genes had a mean survival time of 88 months (95% CI = 72–104 months), those whose tumors overexpressed two of the three genes had a mean survival time of 99 months (95% CI = 78–121 months), while those whose tumors overexpressed all three genes had a mean survival time of 77 months (95% CI = 63–91 months). Increasing incidence of overexpression of these three genes resulted in significantly shorter survival in these patients (Kaplan-Meier analysis, *p* = 0.01; Figure 4B). Cox-regression analysis using tumors that overexpressed none of the three genes as reference revealed that patients whose tumors overexpressed one (HR = 1.675, 95% CI = 0.75–3.741, *p* = 0.203), two (HR = 1.770, 95% CI = 0.790 = 3.966, *p* = 0.166) and all of the three genes (HR = 2.807, 95% CI = 1.482–5.317, *p* = 0.002) had an increased risk for disease progression (Figure 4D).

Indeed, patients whose tumors expressed TAZ, *AXL* and *CTGF* mRNA at low levels (GSE14333: mean survival = 117 months, 95% CI = 110–123; GSE17538: mean survival = 108 months, 95% CI = 95–121 months) had superior survival compared to those whose tumors overexpressed all the three genes (GSE14333: mean survival = 96, 95% CI = 84–108, *p*<0.001; GSE17538: mean survival = 91 months, 95% CI = 81–101 months, *p* = 0.037; Figure S1).

Neither the age nor sex of the patients was factor that determined the number of TAZ, AXL and CTGF that overexpressed in either colon cancer patient cohort. TAZ-AXL-CTGF expression was only significantly lower in tumors stage A compared to tumors of other stages (*p* = 0.022), but were not significantly different between other stages in GSE14333. In GSE17538, TAZ-AXL-CTGF expression was not significantly different between AJCC stages or tumor grades. When the patients were stratified by their stage, we found that TAZ-AXL-CTGF expression was still significantly correlated with poor survival in patients with higher stage tumors. In GSE14333, increasing number of genes overexpressed among TAZ, AXL and CTGF was significantly correlated with a shorter survival (HR = 1.414, 95% CI = 1.031–1.938, *p* = 0.032) in patients with stage C tumors. Similar results were obtained in GSE17538, in which, TAZ-AXL-CTGF expression was associated with poorer survival in patients with tumors of higher grade and higher stage. TAZ-AXL-CTGF expression was correlated with poorer survival in both grade B (HR = 1.315, 95% CI = 1.026 = 1.685, *p* = 0.031) and grade C (HR = 1.671, 95% CI = 1.001–2.790, *p* = 0.05) tumors, as well as AJCC stage 3 (HR = 1.694, 95% CI = 1.075–2.670, *p* = 0.023) and stage 4 (HR = 1.415, 95% CI = 1.074–1.866, *p* = 0.014) tumors.

### Identification of genes that are differentially expressed between TAZ-AXL-CTGF-high and low patients

We found that 39 genes, including TAZ, AXL and CTGF, were significantly differentially expressed between TAZ-AXL-CTGF-high and low patients in both datasets. Some of these 39 genes have been shown to be induced by overexpressing YAP/TAZ-TEAD complexes in transfected cells [19], [29], including Akt3, CCDC80, FBN1, FRMD6, MSRB3, MYL9, PTRF, TAGLN, TNS1, VCAN and ZEB1 (Tables 1 and 2). Also co-regulated with TAZ-AXL-CTGF were genes involved in EMT, migration and invasion, colon cancer progression, calcium signaling, angiogenesis, cytoskeleton association, membrane trafficking, focal adhesion, Hippo pathway regulation, and MMPs inhibition (Tables 1 and 2), suggesting that these biological processes may play an important role in TAZ-AXL-CTGF-mediated colon cancer progression.

**Table 1:** Genes that are co-regulated with TAZ-AXL-CTGF expression in GSE14333 colon cancer patient cohort.

GSE14333	Number of TAZ/AXL/CTGF that are overexpressed							
Gene and phenotype associated with aberrant expression	0		1		2		3	
	Mean	95% CI	Mean	95% CI	Mean	95% CI	Mean	95% CI
	Relative expression levels							
**Epithelial-Mesencymal transition**								
**ACTA2**	97	84–111	105	95–114	120	109–132	147	130–164
**ZEB1**	76	62–90	92	83–100	112	102–122	163	141–186
**ZEB2**	21	19–23	28	25–30	29	27–32	37	34–40
**Migration and Invasion**								
DDR2	95	73–117	108	99–117	126	113–139	189	161–217
**FERMT2**	105	69–140	120	107–132	153	135–171	246	203–288
**AKT3**	48	43–52	56	51–60	61	56–65	79	72–85
**VCAN**	469	386–552	671	578–764	728	636–820	1275	1103–1447
**Colon cancer biomarkers**								
**EFEMP2**	140	125–156	184	166–202	208	187–229	300	268–331
**SULF1**	687	558–816	1003	867–1139	1126	984–1267	1764	1579–1949
**TAGLN**	319	208–430	395	347–443	528	435–621	866	705–1026
**Calcium binding/signaling**								
**FBN1**	156	125–188	217	184–249	234	209–260	415	352–478
**CALD1**	84	73–96	98	89–106	106	98–114	156	135–177
**MGP**	66	39–92	84	60–107	106	81–130	301	216–386
**MYL9**	358	200–515	419	361–477	613	475–752	1137	904–1371
**Angiogenesis**								
ANTXR1	42	39–45	50	46–53	51	47–54	62	57–66
**SERPINF1**	412	340–485	595	529–662	681	594–768	1038	882–1194
**Cytoskeleton associated protein**								
**DPYSL3**	44	35–53	58	51–65	59	53–65	101	86–117
**PDLIM3**	38	28–47	43	39–47	56	50–62	86	73–100
**Membrane trafficking**								
**RAB31**	343	283–404	489	427–551	556	480–631	786	696–876
RAB34	88	69–107	126	112–140	158	137–179	226	200–252
**Focal adhesion**								
**TGFB1I1**	205	162–249	260	230–289	314	273–354	459	397–521
**TNS1**	43	38–48	50	46–54	61	55–66	78	69–87
**Hippo pathway related genes**								
AMOTL1	18	15–22	19	18–21	23	20–25	30	26–33
FRMD6	130	104–155	175	153–197	217	188–246	351	300–401
VGLL3	47	30–64	56	49–63	71	62–80	107	91–123
**Tissue inhibitor of MMPs**								
TIMP2	279	234–323	443	393–492	486	433–538	698	632–763
**TIMP3**	264	221–307	352	299–404	417	362–471	563	502–624
**Others**								
CCDC80	53	31–76	65	57–73	82	71–92	155	128–182
**COL5A1**	362	309–416	516	447–586	559	488–630	872	753–991
**GEM**	361	267–455	474	418–531	534	484–583	945	800–1090
GLT8D2	123	102–144	167	146–187	184	162–205	274	236–311
MSRB3	86	59–113	104	94–115	129	112–146	196	167–225
**NNMT**	330	265–396	483	414–552	565	473–658	848	731–965
**NXN**	170	135–205	260	201–320	274	234–314	360	321–400
**PTRF**	140	117–163	170	157–182	189	172–205	272	242–303
SFRP2	218	100–337	305	208–402	404	296–511	1142	900–1394

**Table 2:** Genes that are co-regulated with TAZ-AXL-CTGF expression in GSE17538 colon cancer patient cohort.

GSE17538	Number of TAZ/AXL/CTGF that are overexpressed							
Gene and phenotype associated with aberrant expression	0		1		2		3	
	Mean	95% CI	Mean	95% CI	Mean	95% CI	Mean	95% CI
	Relative expression levels							
**Epithelial-Mesencymal transition**								
ACTA2	175	165–184	211	198–224	240	228–252	292	269–315
ZEB1	113	106–121	152	140–165	185	171–200	262	234–290
ZEB2	56	54–59	70	66–75	79	75–84	97	92–101
**Migration and Invasion**								
DDR2	203	190–216	250	232–268	316	293–339	440	392–487
FERMT2	133	121–144	201	179–223	259	232–286	416	357–475
AKT3	80	76–84	94	89–100	109	102–116	127	121–133
VCAN	584	515–652	945	823–1066	1314	1126–1503	2188	1958–2419
**Colon cancer biomarkers**								
EFEMP2	243	230–256	310	282–339	403	364–442	513	476–549
SULF1	726	633–818	1186	1026–1345	1897	1665–2129	2803	2578–3029
TAGLN	670	619–722	855	785–925	1215	1055–1375	1779	1562–1996
**Calcium binding/signaling**								
FBN1	169	152–185	263	231–295	376	334–418	623	544–702
CALD1	162	153–171	201	188–214	234	220–249	303	277–330
MGP	101	91–110	151	132–169	207	179–235	381	308–454
MYL9	448	398–498	643	568–717	1010	830–1190	1678	1340–2016
**Angiogenesis**								
ANTXR1	187	180–194	216	206–226	251	240–261	290	278–302
SERPINF1	656	584–728	1106	815–1396	1315	1150–1480	1937	1708–2165
**Cytoskeleton associated proteins**								
DPYSL3	135	125–146	186	168–204	226	204–248	308	280–336
PDLIM3	68	65–71	84	80–89	99	94–104	139	118–160
**Membrane trafficking**								
RAB31	501	443–559	711	619–804	1021	889–1154	1549	1394–1704
RAB34	227	208–247	313	253–372	347	317–378	497	452–542
**Focal adhesion**								
TGFB1I1	388	358–417	465	412–517	672	599–746	880	796–964
TNS1	157	149–165	185	175–195	221	208–235	256	244–268
**Hippo pathway related genes**								
AMOTL1	106	99–114	133	123–143	147	136–157	167	159–175
FRMD6	202	179–224	309	265–353	429	369–490	756	659–853
VGLL3	45	41–50	67	58–76	91	79–103	153	130–176
**Tissue inhibitor of MMPs**								
TIMP2	706	641–771	988	882–1094	1372	1254–1489	1814	1690–1939
TIMP3	485	431–539	694	620–769	959	838–1080	1389	1269–1508
**Others**								
CCDC80	121	112–131	151	137–165	201	182–221	302	262–343
COL5A1	501	451–552	699	602–795	1066	929–1204	1525	1364–1690
GEM	365	328–401	620	533–708	747	648–846	1177	1049–1305
GLT8D2	177	159–195	246	215–276	351	306–395	523	467–579
MSRB3	175	165–186	222	208–237	284	258–310	403	359–446
NNMT	487	436–539	736	651–822	1074	892–1256	1543	1385–1701
NXN	283	258–308	410	348–472	499	440–559	681	613–748
PTRF	264	246–282	317	293–341	378	344–411	564	513–615
SFRP2	331	210–451	530	383–677	1036	779–1294	2199	1763–2634

**Figure 5:**
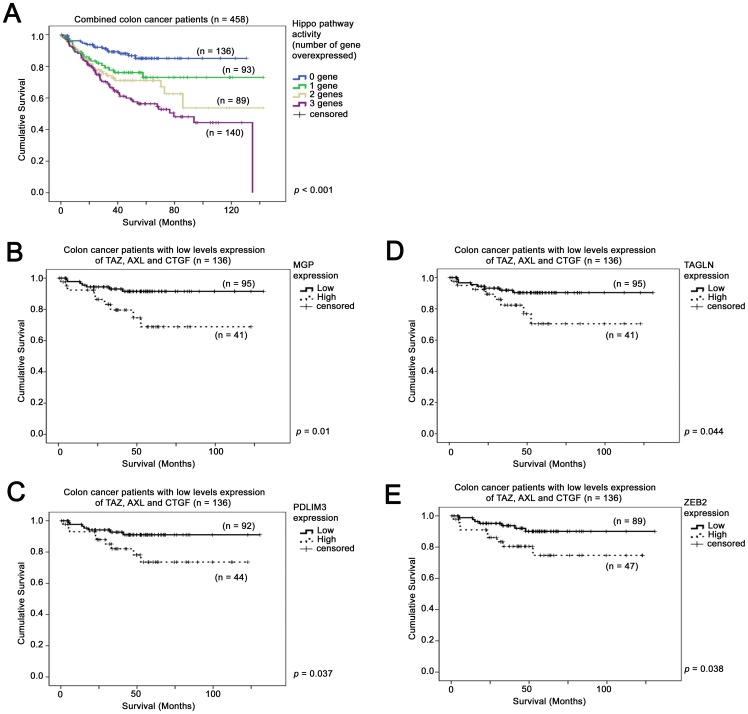
Kaplan-Meier analyses for the mRNA expression of TAZ-AXL-CTGF and its co-regulated genes in the combined colon cancer dataset. (A) Kaplan-Meier analysis for TAZ-AXL-CTGF mRNA expression in the combined colon cancer datasets. Kaplan-Meier analyses for mRNA expression of (B) MGP, (C), PDLIM3, (D) TAGLN and (E) ZEB2 in colon cancer patients expressing low levels of TAZ-AXL-CTGF (blue line in (A)) in the combined colon cancer datasets.

Individually, as expected because of their strong association with the TAZ-AXL-CTGF expressions, all of the genes in the list (Tables 1 and 2) were significantly correlated with patient survival (*p*<0.05; data not shown). More importantly, we found that although none of the genes on the list were associated with patient survival in the TAZ-AXL-CTGF-high group of patients (data not shown), *MGP*, *PDLIM3*, *TAGLN* and *ZEB2* were predictors of survival in the TAZ-AXL-CTGF-low group of patients, in the combined colon cancer patient cohort (Figure 5). When the two colon cancer patient datasets were combined, as expected, TAZ-AXL-CTGF mRNA expression levels were associated with patient survival (*p*<0.001; Figure 5A). A high level of *MGP* (*p* = 0.01; Figure 5B), *PDLIM3* (*p* = 0.037; Figure 5C), *TAGLN* (*p* = 0.044; Figure 5D) and *ZEB2* (*p* = 0.038; Figure 5E) mRNA expression were correlated with a shorter survival time in the TAZ-AXL-CTGF-low group of patients. These results suggest that these four genes may be used as prognostic markers for patients who express low levels of TAZ, *AXL* and *CTGF* mRNA.

### Identification of potential small molecules that could target the 39 gene signature of TAZ-AXL-CTGF co-expression

Analysis to determine alterations in gene expression following small molecule treatment of cancer cells revealed a total of 257 small molecules that were associated with a gene expression signature significantly correlated with the TAZ-AXL-CTGF gene expression signature (the 25 genes that are presented in bold in Tables 1 and 2 together with TAZ, AXL and CTGF). Of the 257 small molecules, 164 had a 100% perturbation stability and 138 of them are inversely correlated with the TAZ-AXL-CTGF gene expression signature. The results are listed in the supplementary information. The top 20 small molecules were further analyzed through a Pubmed search regarding their effects on treatment of colon cancer. We found that amiloride and tretinoin have yielded 55 and 123 publications, respectively, when coupled with colon cancer in the search engine. Several publications have shown their inhibitory effect on colon cancer growth. Amiloride treatment has been shown to inhibit the growth of colon cancer cells *in vitro* [45] and *in vivo* [46]. Importantly, it can sensitize doxorubicin resistant colon cancer cells to treatment with doxorubicin [47], suggesting that amiloride and doxorubicin can be combined to treat doxorubicin resistant colon cancer. Tretinoin, also known as all-trans retinoic acid, has been shown to inhibit proliferation and anchorage-independent growth of colon cancer cells *in vitro* [48], [49] and *in vivo* [50], probably through regulating the differentiation state of cancer cells [51].

### Identification of potential therapeutic targets for patients overexpressing TAZ-AXL-CTGF

**Figure 6:**
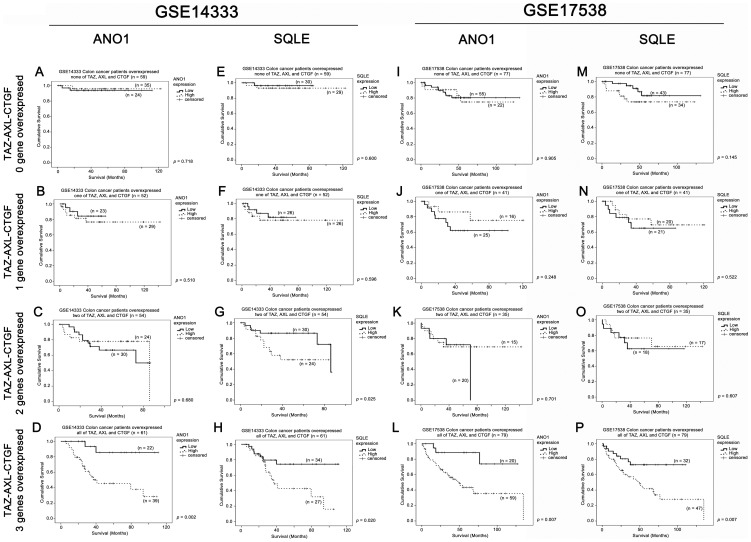
The prognostic significance of *ANO1* and *SQLE* mRNA expression in colon cancer patients stratified by their TAZ-AXL-CTGF mRNA expression. Kaplan-Meier analyses for *ANO1* mRNA expression in patients overexpressing (A) none, (B) one, (C) two and (D) three of the three genes (TAZ, *AXL* and *CTGF*) in the GSE14333 colon cancer patient datasets. Kaplan-Meier analyses for *SQLE* mRNA expression in patients overexpressing (E) none, (F) one, (G) two and (H) three of the three genes in the GSE14333 colon cancer patient datasets. Kaplan-Meier analyses for *ANO1* mRNA expression in patients overexpressing (I) none, (J) one, (K) two and (L) three of the three genes in the GSE17538 colon cancer patient datasets. Kaplan-Meier analyses for *SQLE* mRNA expression in patients overexpressed in the GSE17538 colon cancer patient datasets.

We further investigated which genes could be used to further predict survival in TAZ-AXL-CTGF-high patients; these genes may be potential therapeutic targets specific for this group of patients whose colon cancers are more aggressive. Therefore, we compared gene expression in this group of patients between those patients who were still living and those who were deceased. The two cohorts' top 100 differentially expressed genes (based on survival status) were compared, revealing that *ANO1* and *SQLE* are the two genes commonly differentially expressed between those TAZ-AXL-CTGF-high colon cancer patients who are still alive and those who are deceased. By Kaplan-Meier analysis, we found that these two genes themselves were associated with patient survival in both colon cancer datasets (Figure S2). We then investigated whether the associations between *ANO1* or *SQLE* and patient survival were specific to patients who overexpressed TAZ, *AXL* and *CTGF* (i.e. the TAZ-AXL-CTGF-high subgroup). A low level of *ANO1* expression was significantly (GSE14333, *p* = 0.002; Figure 6D and GSE17538, *p* = 0.007; Figure 6L) associated with better survival in TAZ-AXL-CTGF-high patients in both colon cancer datasets (GSE1433: high level, mean survival = 59 months, 95% CI = 44–73 months, vs. low level, mean survival = 100 months, 95% CI = 86–114 months; GSE17538: high level, mean survival = 66 months, 95% CI = 51–82 months, vs. low level, mean survival = 107 months, 95% CI = 87–127 months), but was not associated with survival in patients with other patterns of expression for TAZ-AXL-CTGF (Figure 6A, B, C, I, J, K). Similarly, a low level of *SQLE* expression was significantly (GSE14333, *p* = 0.02; Figure 6H and GSE17538, *p* = 0.01; Figure 6P) associated with better survival for TAZ-AXL-CTGF-high patients in both colon cancer datasets (GSE1433: high level, mean survival = 55 months, 95% CI = 40–70 months, vs. low level, mean survival = 88 months, 95% CI = 73–103 months; GSE17538: high level, mean survival = 62 months, 95% CI = 45–79 months, vs. low level, mean survival = 86 months, 95% CI = 71–101 months), but was not or less significant in patients with other patterns of expression for TAZ-AXL-CTGF (Figure 6E, F, G, M, N, O). When both *ANO1* and *SQLE* genes were included in multivariate Cox-regression analysis, we found that mRNA expression of *ANO1*, but not *SQLE*, was an independent predictor of patient survival together with both tumor stage and TAZ-AXL-CTGF expression in both colon cancer patient datasets (Tables 3 and 4). These results suggest that *ANO1* and *SQLE* mRNA expression may determine the aggressiveness of TAZ-AXL-CTGF-high tumors and that *ANO1* mRNA expression could be used in combination with TAZ-AXL-CTGF and tumor stage for better prognostification. Our results also imply that blockade of *ANO1* or *SQLE* mRNA expression or inhibition of these two proteins in this group of patients may prolong survival.

**Table 3:** Cox-regression analysis of GSE14333 datasets.

Clinicopathological variables	Multivariate analysis (Forward conditional)	
	Hazard ratio (95% CI)	p-value
Stage (n = 226)	Overall	**0.001**
A (n = 41)	1	Reference
B (n = 94)	2.648 (0.592–11.843)	0.203
C (n = 91)	7.213 (1.688–30.817)	**0.008**
Hippo pathway activity (n = 226)		0.086
0 gene overexpressed (n = 59)	1	Reference
1 gene overexpressed (n = 52)	3.798 (1.009–14.302)	**0.049**
2 genes overexpressed (n = 54)	4.961 (1.428–17.236)	**0.012**
3 genes overexpressed (n = 61)	4.505 (1.310–15.492)	**0.017**
*ANO1* (n = 226)	1.550 (1.161–2.069)	**0.003**

**Table 4:** Cox-regression analysis of GSE17538 datasets.

Clinicopathological variables	Multivariate analysis (Forward conditional)	
	Hazard ratio (95% CI)	p-value
Age (n = 213)	1.024 (1.002–1.046)	**0.029**
Stage (n = 213)	Overall	**<0.001**
1 (n = 27)	1	Reference
2 (n = 65)	4.089 (0.513–32.586)	0.184
3 (n = 70)	6.898 (0.887–53.641)	0.065
4 (n = 51)	79.966 (10.374–616.406)	**<0.001**
Hippo pathway activity (n = 213)		**0.013**
0 gene overexpressed (n = 64)	1	Reference
1 gene overexpressed (n = 38)	1.106 (0.439–2.787)	0.830
2 genes overexpressed (n = 34)	1.050 (0.410–2.691)	0.920
3 genes overexpressed (n = 77)	2.784 (1.208–6.419)	**0.016**
ANO1 (n = 213)	1.855 (1.286–2.677)	**0.001**

### The role of TAZ in colon cancer progression in vitro and in vivo

**Figure 7:**
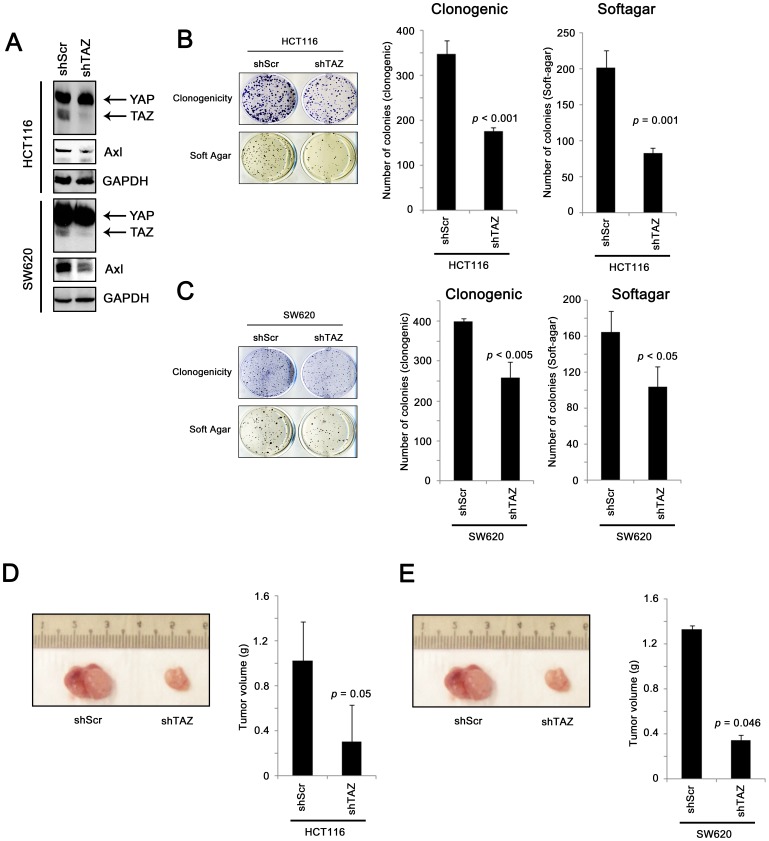
In vitro and in vivo assays for colon cancer cells expressing scramble shRNA or TAZ shRNA. (A) Western blot showing that shTAZ specifically knockdown TAZ, but not YAP, and that AXL was down-regulated in shTAZ cells compared to shScr cells. (B) The clonogenicity and non-adherent growth of HCT116 cells expressing shScr or shTAZ were assessed and the number of colonies formed from three repeats was recorded (C) The clonogenicity and non-adherent growth of SW620 cells expressing shScr or shTAZ were assessed and the number of colonies formed from three repeats was recorded. (D) The in vivo tumorigenicity of HCT116 cells expressing shScr or shTAZ was assessed in nude nice, and the tumor formed was excised and weighted (n = 3 in each group). (E) The in vivo tumorigenicity of SW620 cells expressing shScr or shTAZ was assessed in nude nice, and the tumor formed was excised and weighted (n = 3 in each group).

To confirm the bioinformatics analysis, we investigated the effect of TAZ knockdown in two colon cancer cell lines, HCT116 and SW620. TAZ knockdown in these two cell lines abolished the expression of TAZ and down-regulated AXL expression (Figure 7A). This result is in line with our finding in human specimens showing that AXL is a downstream target of TAZ. Knockdown of TAZ also resulted in a significant reduction in the number of colonies formed in both clonogenic and non-adherent soft-agar assays in these two cell lines (Figure 7B and C). Importantly, these *in vitro* results were replicated in *in vivo* tumorigenesis assay. Both HCT116-shTAZ and SW620-shTAZ cells formed significantly larger tumors in nude mice compared to HCT116-shScr and SW620-shScr cells, respectively (Figure 7D and 7E, respectively). Our results suggest that TAZ expression is required for a higher cell proliferation, non-adherent growth and tumorigenesis in colon cancer cells, traits that are associated with colon cancer progression.

## Discussion

In the present study, we have shown that TAZ mRNA expression is positively correlated with two of its downstream targets, *AXL* and *CTGF*, and that TAZ is significantly associated with poor survival of colon cancer patients in two independent colon cancer datasets, comprising 522 patients. Interestingly, the upregulation of *AXL* and *CTGF*, which reflects the increased transcriptional activity of TAZ-TEAD complexes, can be used in combination with TAZ mRNA expression, for better prognostification in these two independent colon cancer patient datasets. Genes that are co-regulated with TAZ-AXL-CTGF overexpression are involved in several important cellular processes, including cell migration, angiogenesis and calcium signaling, as well as others that have already been described as prognostic markers for colon cancer progression. These genes may be upstream factors or downstream effectors of TAZ and the dysregulated Hippo pathway in colon cancers. Importantly, we also identified two potential therapeutic targets, *ANO1* and *SQLE*, for patients with upregulated TAZ-AXL-CTGF expression; downregulation of either of these two genes may greatly improve the survival of TAZ-AXL-CTGF-high colon cancer patients.

An increase in TAZ mRNA may not necessarily correlate with an increase of its transcriptional activity, due to the fact that TAZ could be post-translationally regulated via cytoplasmic sequestration by 14-3-3 [1]. Possible disparity between TAZ mRNA expression and its transcriptional activity may impair the lone utilization of TAZ mRNA expression as a prognostic marker. Previously, we have shown that *AXL* [43] and *CTGF* [44] are both downstream targets of the Hippo pathway. In the present study, we have analyzed the transcriptional outcome of TAZ-TEAD complexes in colon cancer using AXL or CTGF alone or in combination with TAZ and found that patients who co-overexpressed all three genes had significantly poorer survival compared to those who had other expression patterns. Our results, therefore, strongly suggest that TAZ, *AXL* and *CTGF* can be used in combination for prognostification in colon cancer patients.

Several genes that are related to EMT are overexpressed in high TAZ-AXL-CTGF expressing patients (Tables 1 and 2). These include *ACTA2* [52], *ZEB1* [53], [54] and *ZEB2* [55], [56]. Interestingly, *ZEB1* has been shown to be a downstream target of TAZ in retinal pigment epithelial cells [57], suggesting that *ZEB1* may act as a downstream effector of TAZ to promote cancer metastasis, while *ZEB1* and *ZEB2* have also been shown to be prognostic markers in colon cancer [53], [56]. Genes that govern migration and invasion were also differentially expressed (Tables 1 and 2). *AKT3*, which has been shown to contribute oncogenic functions similar to other AKT isoforms [58], was up-regulated in the TAZ-AXL-CTGF-high group of patients. *DDR2* [59], [60], *FERMT2* [61], [62], [63] and *VCAN* [64] also play significant roles in cell adhesion and migration and are upregulated in TAZ-AXL-CTGF-high group of patients.

Colon cancer biomarkers were also differentially expressed in these two groups of patients (Tables 1 and 2), namely *EFEMP2*, a serum biomarker for early detection of colon cancer [65] and *SULF1*, a protein important in colon cancer diagnosis [66] and whose serum level is elevated in patients with colon adenomas [67]. Genes that are implicated in angiogenesis were also identified (Tables 1 and 2); both *VCAN* [68], [69] and *ANTXR1* [70], [71], [72] promote angiogenesis for cancer progression. Four genes that are involved in calcium binding or signaling were co-regulated with TAZ-AXL-CTGF in the colon cancer specimens, including *FBN1* [73], *CALD1* [74], *MGP* [75] and *MYL9* [76].

Interestingly, genes that play a role in regulating the Hippo pathway were also co-regulated with TAZ-AXL-CTGF. The angiomotin family members act as tumor suppressors by inhibiting the oncogenic functions of YAP and TAZ [44], [77], while *FRMD6* also acts as an antagonist of YAP by activating Hippo pathway kinases [78]. In this study, we found that *AMOLT1* and *FRMD6* are co-overexpressed in TAZ-AXL-CTGF positive tumors, suggesting that *AMOLT1* and *FRMD6* may form a negative regulatory loop with TAZ activation, which requires further investigation *in vitro* in colon cancer cell line models. In addition, VGLL3, which has been shown to act as a co-activator of TEAD transcription factors is also upregulated in TAZ-AXL-CTGF-high tumors [79].

Treatments targeting TAZ-AXL-CTGF-high cancers are required due to the aggressive nature of this type of colon cancer. We employed two different analyses to identify potential therapeutic agents and gene targets for this type of cancer. In sccMap, we found that amiloride and tretinoin may inhibit the gene expression signature associated with this aggressive type of colon cancer; these molecules have been shown in the literature to provide strong inhibitory effects on colon cancer proliferation *in vitro* and *in vivo*. The other 18 small molecules of the top twenty identified by sccMap as listed in the supplementary information have yet to be studied in term of their effect on colon cancer progression driven by the overexpression of TAZ-AXL-CTGF co-overexpression.

*ANO1*, also named DOG1, has been shown to be ubiquitously expressed in gastrointestinal stromal tumors [80] and its overexpression is correlated with development of distant metastases in head and neck squamous cell carcinoma [81]. Recently, *ANO1* was shown to promote tumorigenesis and cancer progression via activating MAPK [82]. In the same study, the authors demonstrated that pharmacological inhibition of *ANO1* resulted in cancer cell death, however the role of *ANO1* in colon cancer progression has not been examined. In the present study, we found that a low level expression of *ANO1* in the aggressive TAZ-AXL-CTGF-high subgroup of colon cancer patients is associated with prolonged survival (high level to low level of *ANO1* expression; mean survival from 59 to 100 months and from 66 to 107 month in GSE14333 and GSE17538 patient datasets, respectively), suggesting that pharmacological inhibition of *ANO1* may represent a novel therapeutic approach for this group of patients with aggressive colon cancer. *ANO1* is a calcium ion-activated chloride channel. Interestingly, multiple genes involved in calcium signaling (*FBN1*, *CALD1*, *MGP* and *MYL9*) were co-overexpressed with TAZ-AXL-CTGF in colon cancer. Due to the fact that calcium signaling plays an important role in cancer progression [83], [84], [85], further investigation into the relationship between *ANO1* and calcium signaling in TAZ-AXL-CTGF-mediated cancer progression is warranted. Nonetheless, our results provide a clue to the involvement of calcium signaling and Ca^2+^-activated CI^−^ channels in colon cancer progression mediated by the Hippo pathway.

Little is known about how *SQLE* promotes cancer progression. It has been shown to be overexpressed in lung squamous cell carcinoma by suppression subtractive hydridization [86], in pancreatic cancer by genome-wide analysis using microarray based techniques [87], and in prostate cancer progression by bioinformatics analysis [88]. Its overexpression in breast cancer is correlated with decreased distant metastasis-free survival [89]. These results show that *SQLE* promotes cancer progression in multiple types of cancer. Again, its role in colon cancer progression was undefined. In the present study, we also found that a high level expression of *SQLE* is associated with poorer survival in TAZ-AXL-CTGF-high patients to be associated with poorer survival (high level to low level of SQLE expression; mean survival from 55 to 88 months and from 62 to 102 month in GSE14333 and GSE17538 patient datasets, respectively), suggesting that SQLE may be a novel therapeutic target for this group of patients.

In conclusion, this study has shown that TAZ-AXL-CTGF in combination may be a novel prognostic indicator for colon cancer progression, and that their overexpression is associated with increased expression of genes that are associated with colon cancer progression. Furthermore, *ANO1* and *SQLE* overexpression may further define a poorer prognosis for colon cancer patients overexpressing TAZ-AXL-CTGF.

## Supporting Information

Figure S1 **Colon cancer patients expressing low levels of TAZ, AXL and CTGF had superior survival.** Patients were stratified into two groups; those whose tumors expressed TAZ, AXL and CTGF mRNA at low level (solid line) and those whose tumors expressed at least one of TAZ, AXL and CTGF at high level (dotted line). Kaplan-Meier analyses for these two subgroups of patients in (A) GSE14333 and (B) GSE17538 colon cancer datasets.
(TIF)

Figure S2 **The associations between ANO1 or SQLE, and survival in colon cancer patients.** Kaplan-Meier analyses for (A) *ANO1* and (B) *SQLE* mRNA expression in the GSE14333 colon cancer patient dataset. Kaplan-Meier analyses for (C) *ANO1* and (D) *SQLE* mRNA expression in the GSE17538 colon cancer patient dataset.
(TIF)
