# Correction: TAZ Expression as a Prognostic Indicator in Colorectal Cancer

**DOI:** 10.1371/journal.pone.0250187

**Published:** 2021-04-08

**Authors:** Hiu-Fung Yuen, Cian M. McCrudden, Yu-Han Huang, Jill M. Tham, Xiaoqian Zhang, Qi Zeng, Shu-Dong Zhang, WanJin Hong

Following publication of this article [1], concerns were raised about duplication of panels in Figs 1 and 7, and S2 Fig. Here the authors provide corrected figures to address errors in figure preparation.

**Fig 1:**
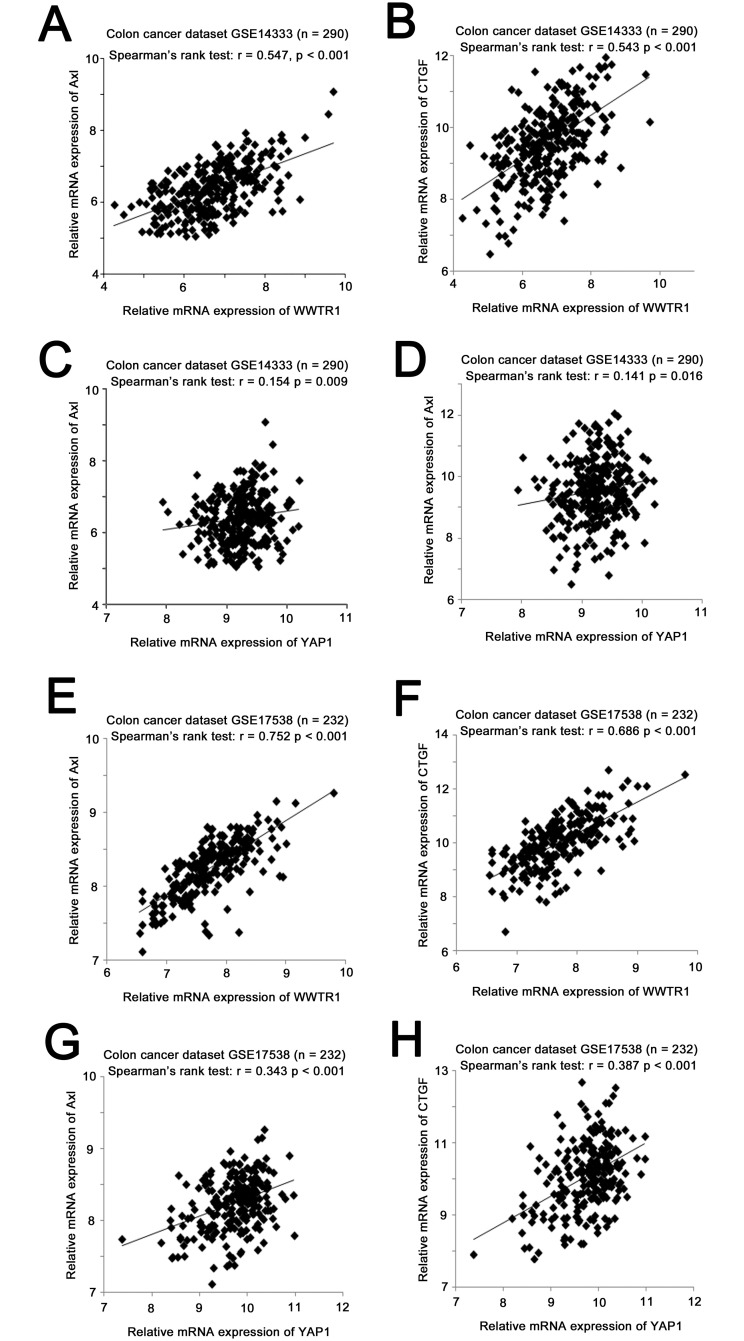
The correlations among mRNA expression of TAZ (WWTR1), YAP (YAP1), AXL and CTGF in colon cancer specimens. Scatter plots for (A) TAZ mRNA expression versus *AXL* mRNA expression, (B) TAZ mRNA expression versus CTGF mRNA expression, (C) YAP mRNA expression versus *AXL* mRNA expression, and (D) YAP mRNA expression versus CTGF mRNA expression in the GSE14333 colon cancer datasets, and (E) TAZ mRNA expression versus *AXL* mRNA expression, (F) TAZ mRNA expression versus CTGF mRNA expression, (G) YAP mRNA expression versus *AXL* mRNA expression, and (H) YAP mRNA expression versus CTGF mRNA expression in the GSE17538 colon cancer datasets.

**Fig 7:**
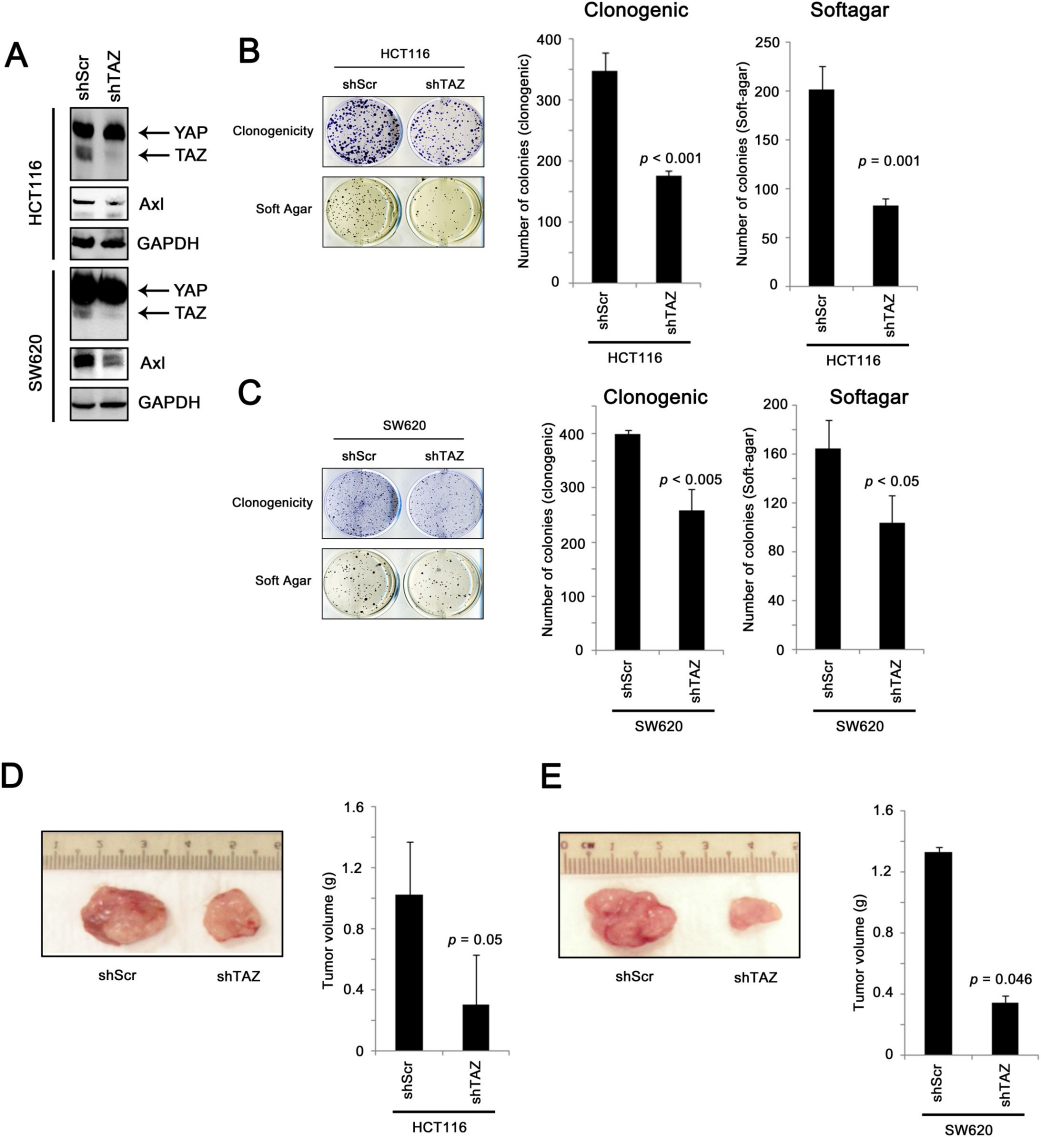
In vitro and in vivo assays for colon cancer cells expressing scramble shRNA or TAZ shRNA. (A) Western blot showing that shTAZ specifically knockdown TAZ, but not YAP, and that AXL was down-regulated in shTAZ cells compared to shScr cells. (B) The clonogenicity and non-adherent growth of HCT116 cells expressing shScr or shTAZ were assessed and the number of colonies formed from three repeats was recorded (C) The clonogenicity and non-adherent growth of SW620 cells expressing shScr or shTAZ were assessed and the number of colonies formed from three repeats was recorded. (D) The in vivo tumorigenicity of HCT116 cells expressing shScr or shTAZ was assessed in nude nice, and the tumor formed was excised and weighted (n = 3 in each group). (E) The in vivo tumorigenicity of SW620 cells expressing shScr or shTAZ was assessed in nude nice, and the tumor formed was excised and weighted (n = 3 in each group).

In the originally published Fig 1, the scatter plot in panel 1H is incorrect and is a duplicate of that in panel 1G. Here, the revised Fig 1 is provided in which panel 1H is replaced with the correct scatter plot from the original study data.

The underlying data for the revised Fig 1 is provided as Supporting Information (S1 File).

There is an error in the in-text figure citations in the 7^th^ sentence of the section “TAZ, but not YAP, mRNA expression is a predictor for patient survival” of the Results. The correct sentence is:

“On the other hand, YAP mRNA expression did not significantly correlate with patient survival by Kaplan-Meier analysis (GSE14333: *p* = 0.519; Figure 2E and GSE17538: p = 0.634; Figure 2F) or by Cox-regression analysis (GSE14333: p = 0.673; Figure 2G and GSE17538: p = 0.979; Figure 2H) in either dataset.”

The authors provide the following additional clarification: In the originally published Figure 2G, the results for the univariate analysis are the same as the results for the multivariate analysis because there is only one variable (the Stage) being retained in the Cox regression model after the stepwise multivariate analysis.

In the originally published Fig 7, the photograph in panel 7E is incorrect and is a duplicate of that in panel 7D. The original image was modified for presentation purposes to splice out other replicates and show the representative tumors adjacent to the tape measure. Here the revised Fig 7 is provided in which the photographs in both panels D and E show tumors from the correct groups using the replicates that appear adjacent to the tape measure in the underlying photograph.

Photographs showing tumors from the three replicates in each group and the tumor volume (g) data underlying the charts in Fig 7D–7E are provided as Supporting Information (S2 File).

In the originally published S2 Fig, the Kaplan-Meier analyses in panels S2C and S2D are incorrect, and the chart in panel S2C is a duplicate of that in S2A. Here the revised S2 Fig is provided in which panels S2C-D are replaced with the correct charts from the original study data.

The underlying data for S2 Fig are provided as Supporting Information (S3 File).

The underlying data for the Figure parts discussed above are provided as Supporting Information (S1–S3 Files). Underlying data for the rest of the article are available upon request from the corresponding author.

## Supporting information

S2 Fig The associations between ANO1 or SQLE, and survival in colon cancer patients. Kaplan-Meier analyses for (A) *ANO1* and (B) *SQLE* mRNA expression in the GSE14333 colon cancer patient dataset. Kaplan-Meier analyses for (C) *ANO1* and (D) *SQLE* mRNA expression in the GSE17538 colon cancer patient dataset.
(TIF)

S1 File Underlying data for Fig 1. (XLSX)

S2 File Underlying data for Fig 7. (PDF)

S3 File Underlying data for S2 Fig. (ZIP)
