# Supplementary figures and images for: TAZ Expression as a Prognostic Indicator in Colorectal Cancer
Source: PLoS One. 2013 Jan 23;8(1):e54211. doi: 10.1371/journal.pone.0054211 (PMC3553150; doi:10.1371/journal.pone.0054211)

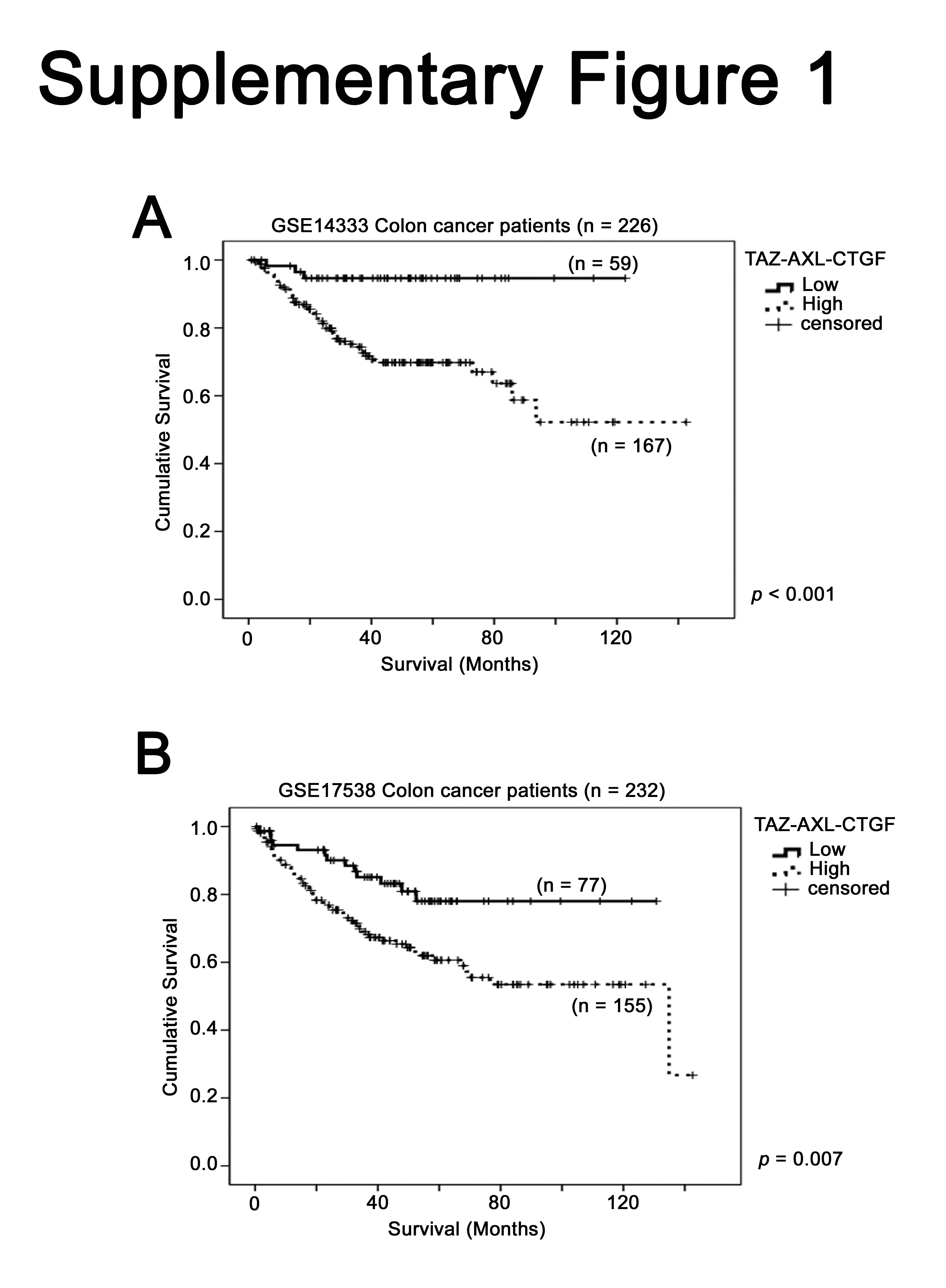

Supplement: Figure S1 — Colon cancer patients expressing low levels of TAZ, AXL and CTGF had superior survival. Patients were stratified into two groups; those whose tumors expressed TAZ, AXL and CTGF mRNA at low level (solid line) and those whose tumors expressed at least one of TAZ, AXL and CTGF at high level (dotted line). Kaplan-Meier analyses for these two subgroups of patients in (A) GSE14333 and (B) GSE17538 colon cancer datasets. (TIF) [file pone.0054211.s001.tif]

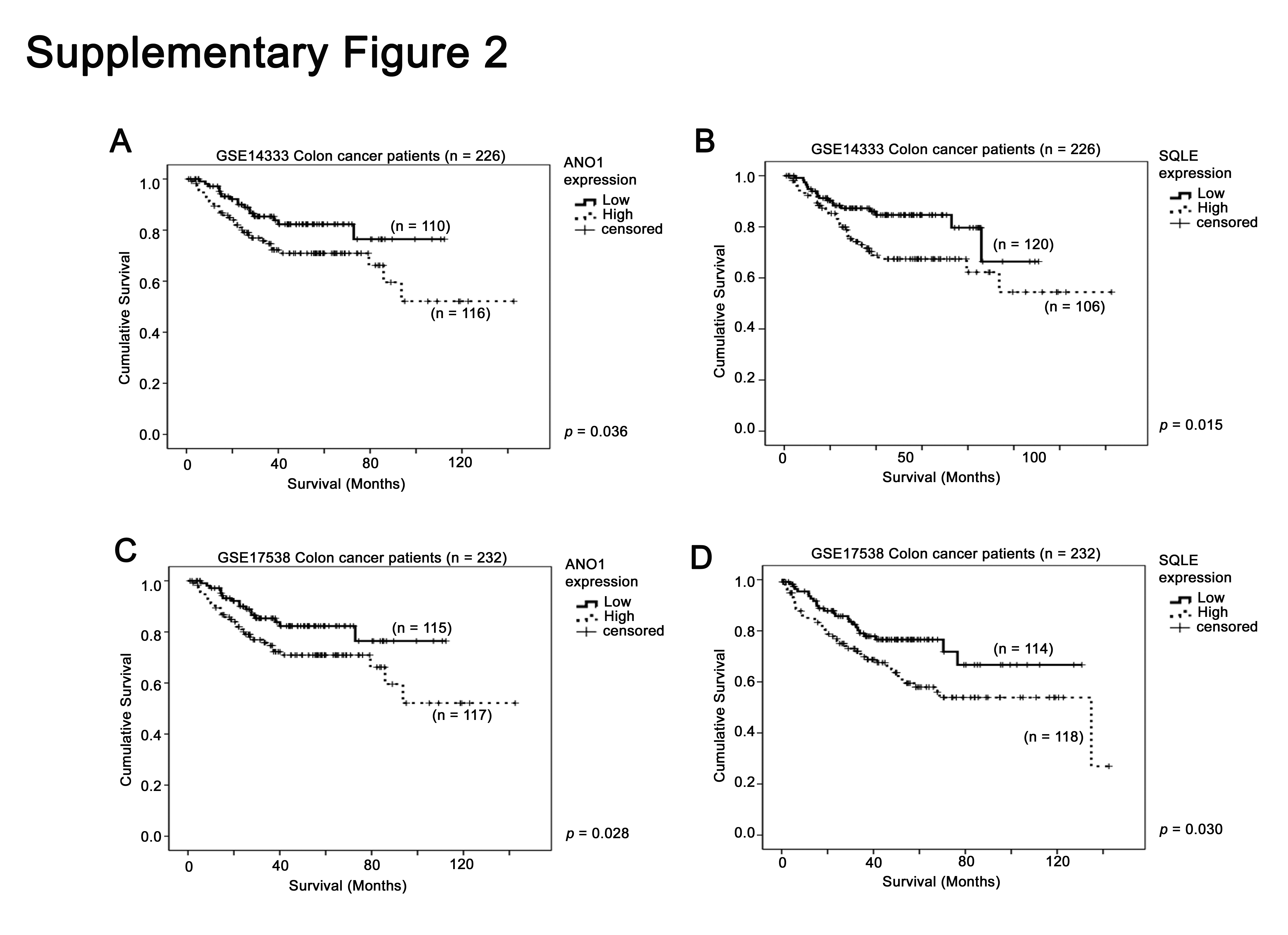

Supplement: Figure S2 — The associations between ANO1 or SQLE, and survival in colon cancer patients. Kaplan-Meier analyses for (A) ANO1 and (B) SQLE mRNA expression in the GSE14333 colon cancer patient dataset. Kaplan-Meier analyses for (C) ANO1 and (D) SQLE mRNA expression in the GSE17538 colon cancer patient dataset. (TIF) [file pone.0054211.s002.tif]
