# Supplementary figures and images for: Correction: TAZ Expression as a Prognostic Indicator in Colorectal Cancer
Source: PLoS One. 2021 Apr 8;16(4):e0250187. doi: 10.1371/journal.pone.0250187 (PMC8031417; doi:10.1371/journal.pone.0250187)

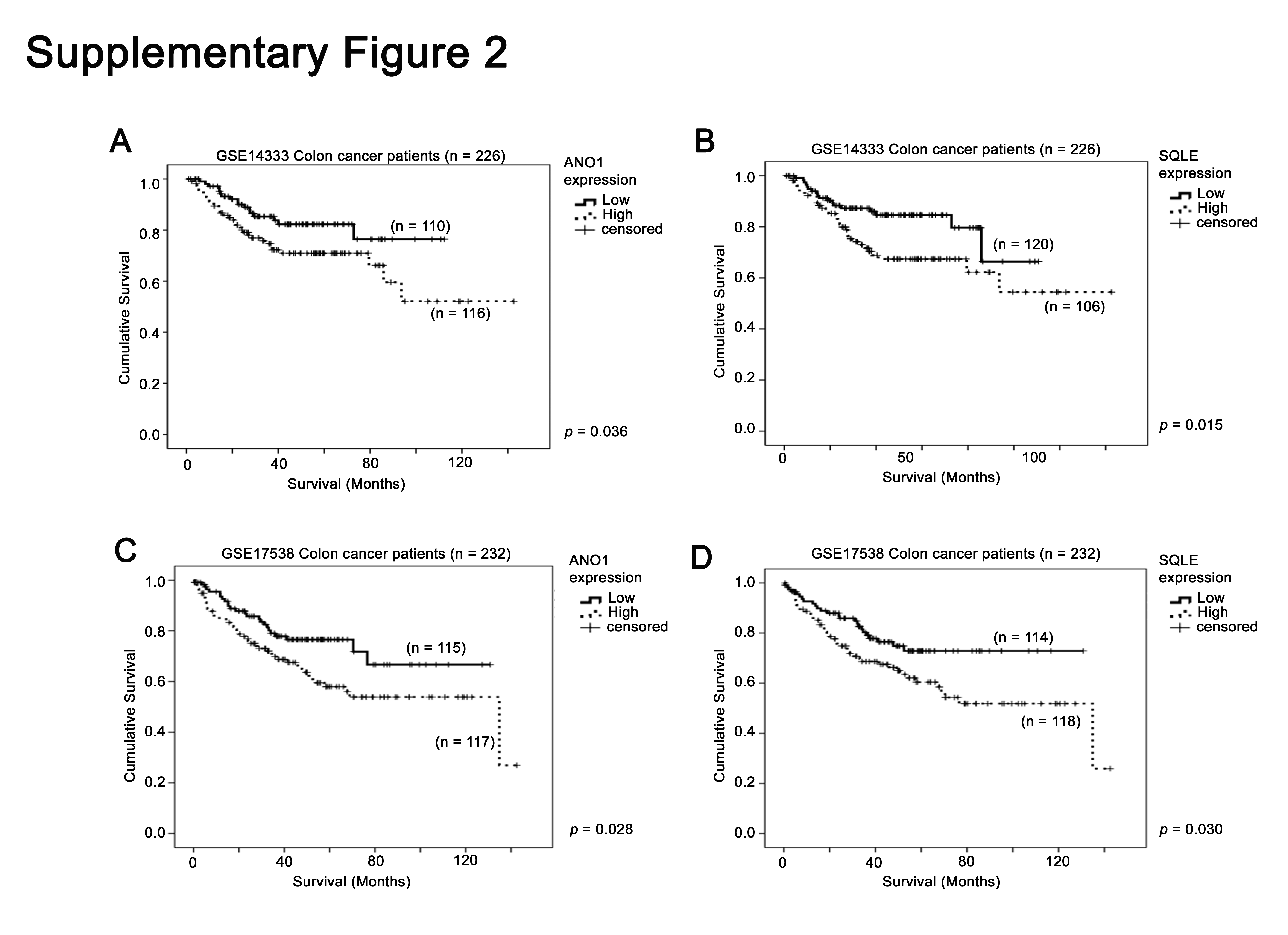

Supplement: S2 Fig — Kaplan-Meier analyses for (A) ANO1 and (B) SQLE mRNA expression in the GSE14333 colon cancer patient dataset. Kaplan-Meier analyses for (C) ANO1 and (D) SQLE mRNA expression in the GSE17538 colon cancer patient dataset. (TIF) [file pone.0250187.s001.tif]

## HCT116 human colon cancer xenografts in nude mice

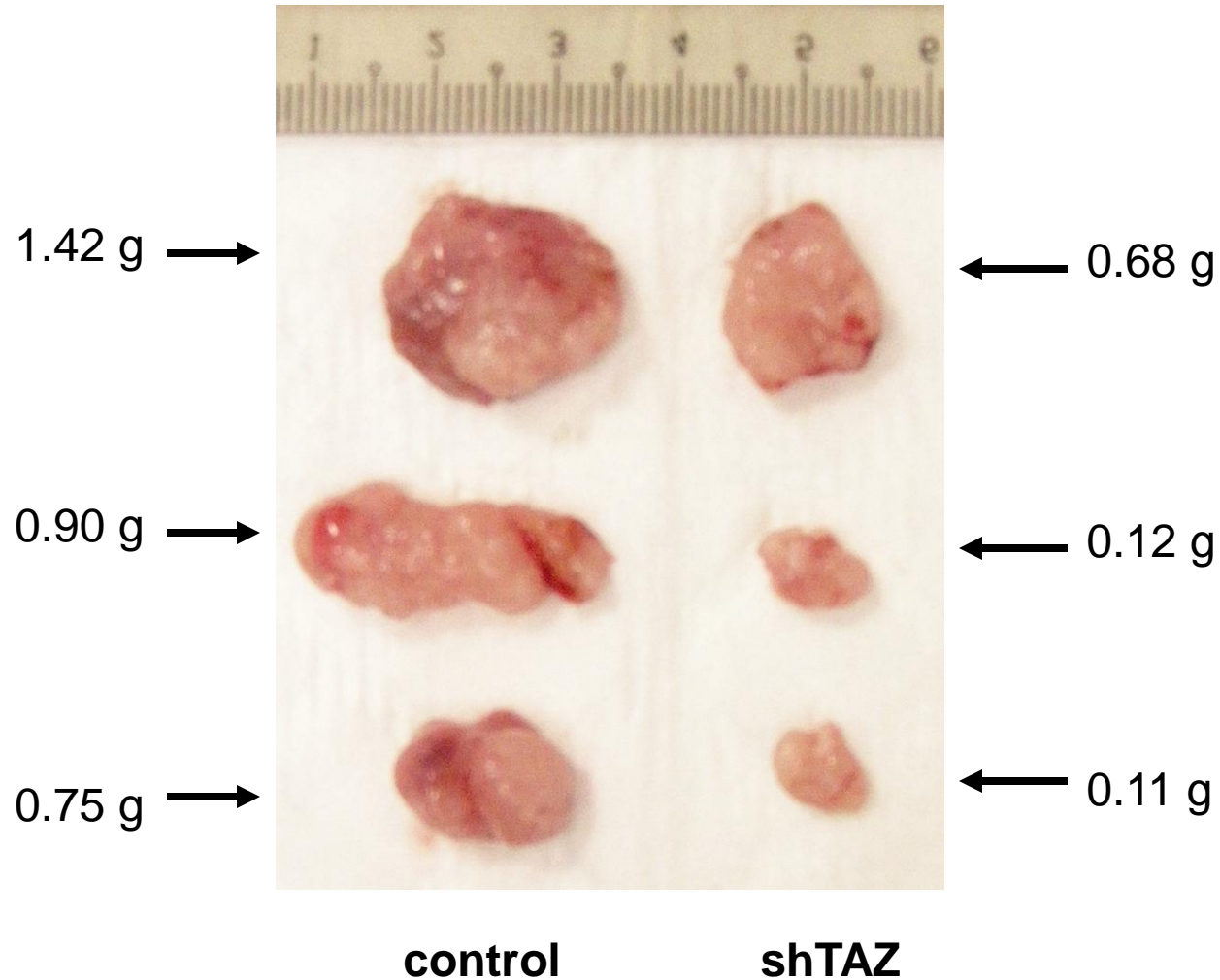

## SW620 human colon cancer xenografts in nude mice

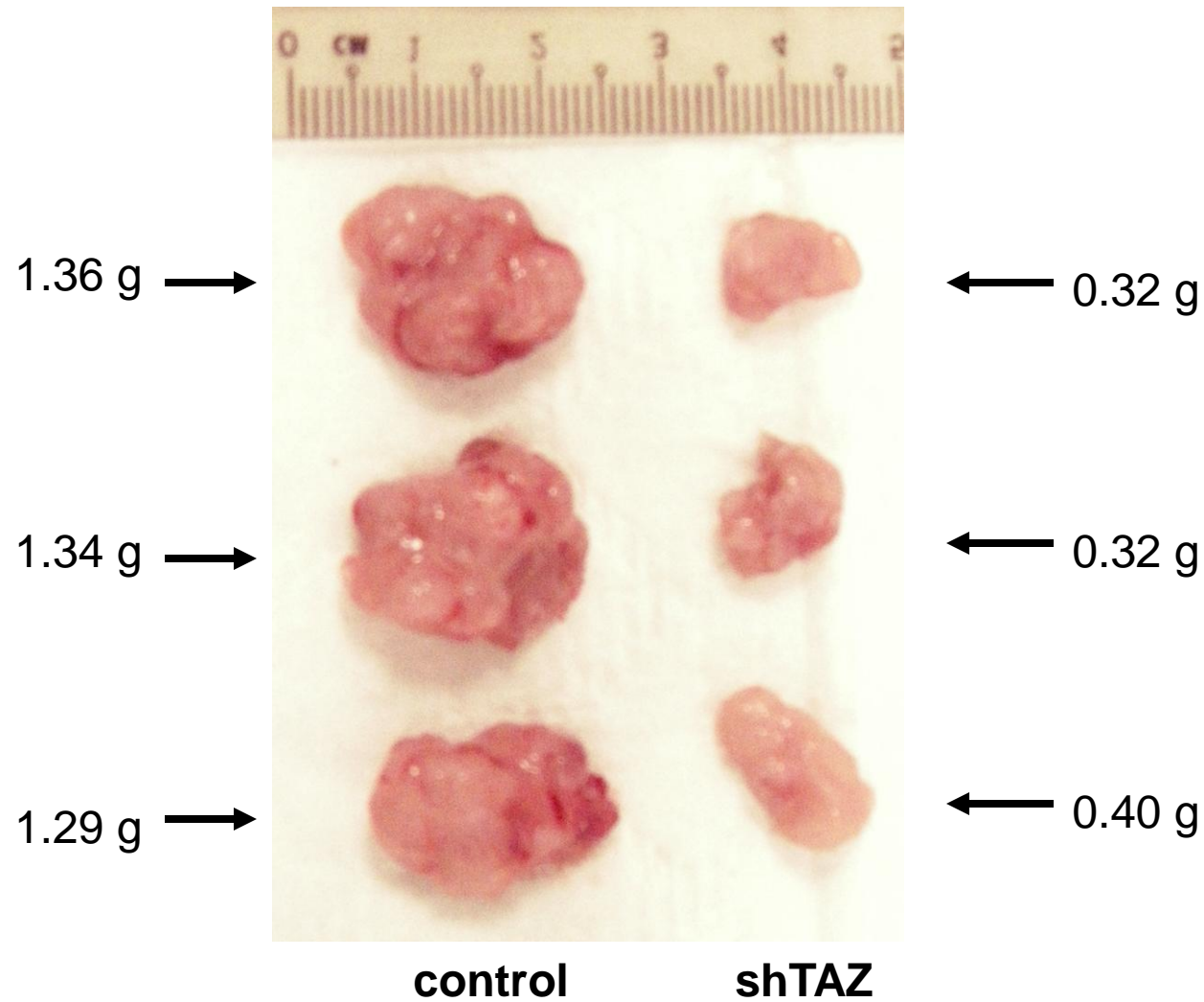

Supplement: S2 File — (PDF) [file pone.0250187.s003.pdf]
