# Supplementary material for: Correction: TAZ Expression as a Prognostic Indicator in Colorectal Cancer
Source: PLoS One. 2021 Apr 8;16(4):e0250187. doi: 10.1371/journal.pone.0250187 (PMC8031417; doi:10.1371/journal.pone.0250187)

```
GET
  FILE='C:\Users\hfyuen\Desktop\Axl-WWTR-CTGF-YAP1 in colon cancer\Threapeutic targets\GSE14
DATASET NAME DataSet1 WINDOW=FRONT.
GET
  FILE='C:\Users\hfyuen\Desktop\Axl-WWTR-CTGF-YAP1 in colon cancer\Threapeutic targets\GSE17
DATASET NAME DataSet2 WINDOW=FRONT.
DATASET ACTIVATE DataSet1.
KM Survival_time BY ANO1_HL
  /STATUS=Survival_Status(2)
  /PRINT MEAN
  /PLOT SURVIVAL
  /TEST LOGRANK BRESLOW TARONE
  /COMPARE OVERALL POOLED.
```

Kaplan-Meier

Notes

Output Created		07-Jun-2012 09:37:34
Comments		
Input	Data	C:\Users\hfyuen\Desktop\Axl-WWTR-CTGF-YAP1 in colon cancer\Threapeutic targets\GSE14333_all hippo_coexpressed_therapeutics genes.sav
	Active Dataset	DataSet1
	Filter	<none>
	Weight	<none>
	Split File	<none>
	N of Rows in Working Data File	290
Missing Value Handling	Definition of Missing	User-defined missing values are treated as missing.
	Cases Used	Statistics are based on all cases with valid data for all variables in the analysis.
Syntax		KM Survival_time BY ANO1_HL /STATUS=Survival_Status(2) /PRINT MEAN /PLOT SURVIVAL /TEST LOGRANK BRESLOW TARONE /COMPARE OVERALL POOLED.
Resources	Processor Time	00 00:00:01.856
	Elapsed Time	00 00:00:03.274

[DataSet1] C:\Users\hfyuen\Desktop\Axl-WWTR-CTGF-YAP1 in colon cancer\Threapeutic targets\GSE14333_all hippo_coexpressed_therapeutics genes.sav

Case Processing Summary

AN01_HL	Total N	N of Events	Censored	
			N	Percent
1.00	110	17	93	84.5%
2.00	116	33	83	71.6%
Overall	226	50	176	77.9%

Means and Medians for Survival Time

AN01_HL	Mean[a]				Median	
	Estimate	Std. Error	95% Confidence Interval		Estimate	Std. Error
			Lower Bound	Upper Bound		
1.00	94.079	4.179	85.889	102.269	.	.
2.00	96.235	7.183	82.156	110.315	.	.
Overall	105.001	5.268	94.676	115.326	.	.

Means and Medians for Survival Time

AN01_HL	Median	
	95% Confidence Interval	
	Lower Bound	Upper Bound
1.00	.	.
2.00	.	.
Overall	.	.

a. Estimation is limited to the largest survival time if it is censored.

Overall Comparisons

	Chi-Square	df	Sig.
Log Rank (Mantel-Cox)	4.383	1	.036
Breslow (Generalized Wilcoxon)	4.546	1	.033
Tarone-Ware	4.419	1	.036

Test of equality of survival distributions for the different levels of AN01_HL.

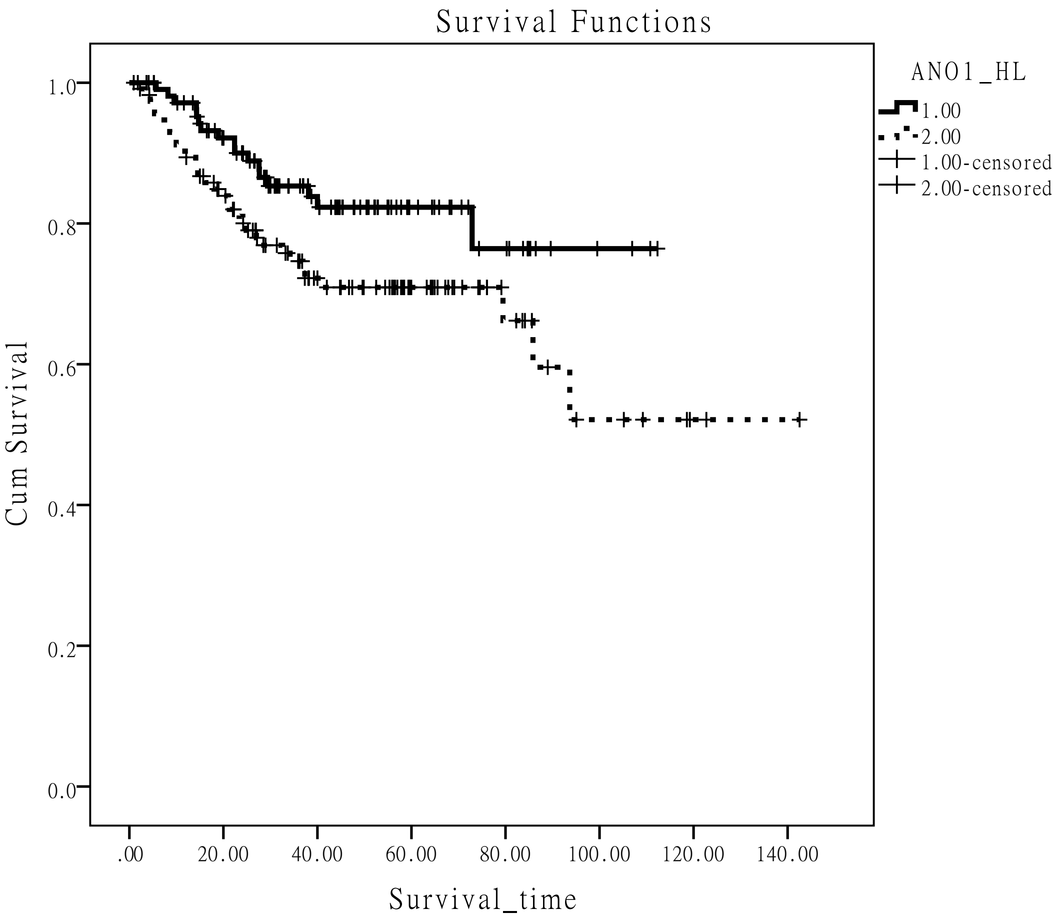


```
KM Survival_time BY SQLE_HL
  /STATUS=Survival_Status(2)
  /PRINT MEAN
  /PLOT SURVIVAL
  /TEST LOGRANK BRESLOW TARONE
  /COMPARE OVERALL POOLED.
```

Kaplan-Meier

Notes

Output Created		07-Jun-2012 09:38:45
Comments		
Input	Data	C:\Users\hfyuen\Desktop\Axl-WWTR-CTGF-YAP1 in colon cancer\Threapeutic targets\GSE14333_all hippo_coexpressed_therapeutics genes.sav
	Active Dataset	DataSet1
	Filter	<none>
	Weight	<none>
	Split File	<none>
	N of Rows in Working Data File	290
Missing Value Handling	Definition of Missing	User-defined missing values are treated as missing.
	Cases Used	Statistics are based on all cases with valid data for all variables in the analysis.
Syntax		KM Survival_time BY SQLE_HL<br>/STATUS=Survival_Status(2)<br>/PRINT MEAN<br>/PLOT SURVIVAL<br>/TEST LOGRANK BRESLOW TARONE<br>/COMPARE OVERALL POOLED.
Resources	Processor Time	00 00:00:00.296
	Elapsed Time	00 00:00:00.267

[DataSet1] C:\Users\hfyuen\Desktop\Axl-WWTR-CTGF-YAP1 in colon cancer\Threapeutic targets\GSE14333_all hippo_coexpressed_therapeutics genes.sav

Case Processing Summary

SQLE_HL	Total N	N of Events	Censored	
			N	Percent
1.00	120	18	102	85.0%
2.00	106	32	74	69.8%
Overall	226	50	176	77.9%

Means and Medians for Survival Time

SQLE_HL	Mean[a]				Median	
	Estimate	Std. Error	95% Confidence Interval		Estimate	Std. Error
			Lower Bound	Upper Bound		
1.00	91.386	4.606	82.358	100.415	.	.
2.00	95.892	7.139	81.899	109.885	.	.
Overall	105.001	5.268	94.676	115.326	.	.

Means and Medians for Survival Time

SQLE_HL	Median 95% Confidence Interval Lower Bound	Upper Bound
1.00	.	.
2.00	.	.
Overall	.	.

a. Estimation is limited to the largest survival time if it is censored.

Overall Comparisons

	Chi-Square	df	Sig.
Log Rank (Mantel-Cox)	5.870	1	.015
Breslow (Generalized Wilcoxon)	5.494	1	.019
Tarone-Ware	5.947	1	.015

Test of equality of survival distributions for the different levels of SQLE_HL.

Survival Functions

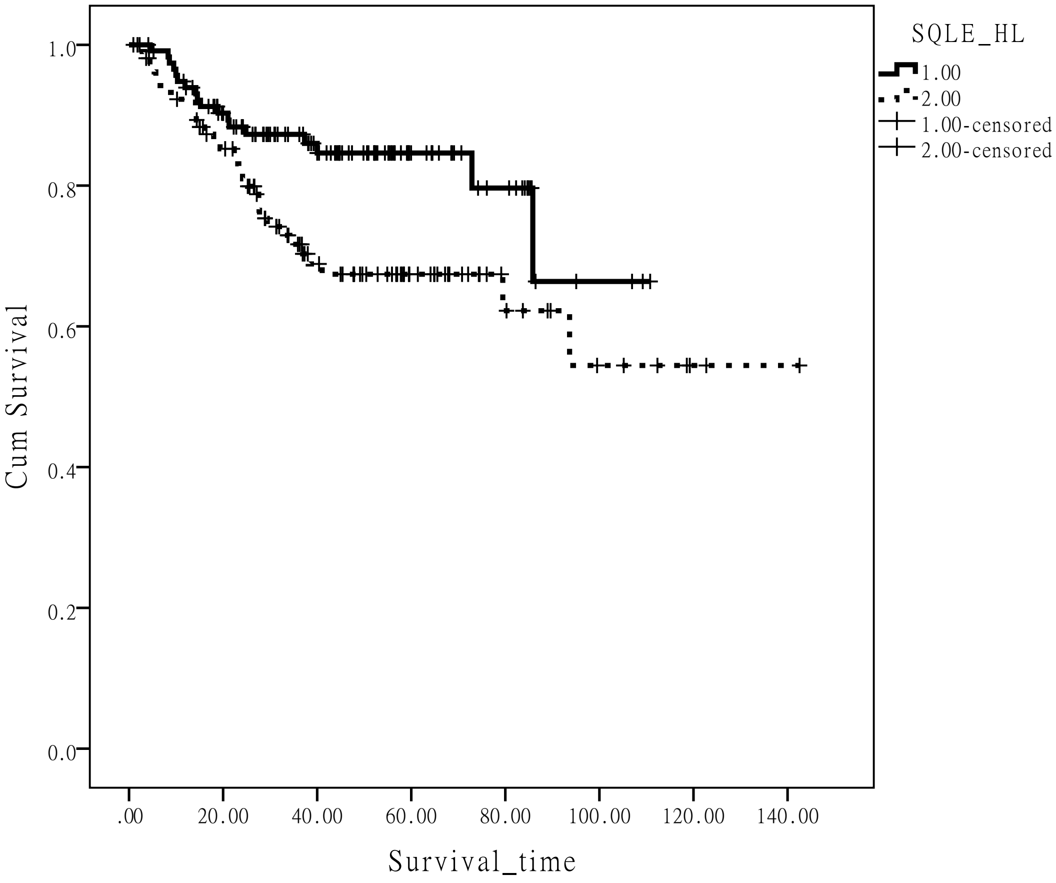

```
 DATASET ACTIVATE DataSet2.
KM DSS_time BY ANO1_HL
  /STATUS=DSS(2)
  /PRINT MEAN
  /PLOT SURVIVAL
  /TEST LOGRANK BRESLOW TARONE
  /COMPARE OVERALL POOLED.
```

Kaplan-Meier

Notes

Output Created		07-Jun-2012 09:39:15
Comments		
Input	Data	C:\Users\hfyuen\Desktop\Axl-WWTR-CTGF-YAP1 in colon cancer\Threapeutic targets\GSE17536 and 537 combined_all hippo_coexpressed_therapeutics genes.sav
	Active Dataset	DataSet2
	Filter	<none>
	Weight	<none>
	Split File	<none>
	N of Rows in Working Data File	238
Missing Value Handling	Definition of Missing	User-defined missing values are treated as missing.
	Cases Used	Statistics are based on all cases with valid data for all variables in the analysis.
Syntax		KM DSS_time BY ANO1_HL /STATUS=DSS(2) /PRINT MEAN /PLOT SURVIVAL /TEST LOGRANK BRESLOW TARONE /COMPARE OVERALL POOLED.
Resources	Processor Time	00 00:00:00.296
	Elapsed Time	00 00:00:00.291

[DataSet2] C:\Users\hfyuen\Desktop\Axl-WWTR-CTGF-YAP1 in colon cancer\Threapeutic targets\GSE17536 and 537 combined_all hippo_coexpressed_therapeutics genes.sav

Case Processing Summary

ANO1_HL	Total N	N of Events	Censored	
			N	Percent
1.00	115	25	90	78.3%
2.00	117	45	72	61.5%
Overall	232	70	162	69.8%

Means and Medians for Survival Time

AN01_HL	Mean[a]				Median	
			95% Confidence Interval			
	Estimate	Std. Error	Lower Bound	Upper Bound	Estimate	Std. Error
1.00	99.298	5.795	87.940	110.656	.	.
2.00	87.699	6.077	75.787	99.610	134.860	44.483
Overall	95.656	4.597	86.647	104.665	134.860	41.419

Means and Medians for Survival Time

AN01_HL	Median	
	95% Confidence Interval	
	Lower Bound	Upper Bound
1.00	.	.
2.00	47.674	222.046
Overall	53.678	216.042

a. Estimation is limited to the largest survival time if it is censored.

Overall Comparisons

	Chi-Square	df	Sig.
Log Rank (Mantel-Cox)	4.823	1	.028
Breslow (Generalized Wilcoxon)	4.816	1	.028
Tarone-Ware	4.938	1	.026

Test of equality of survival distributions for the different levels of AN01_HL.

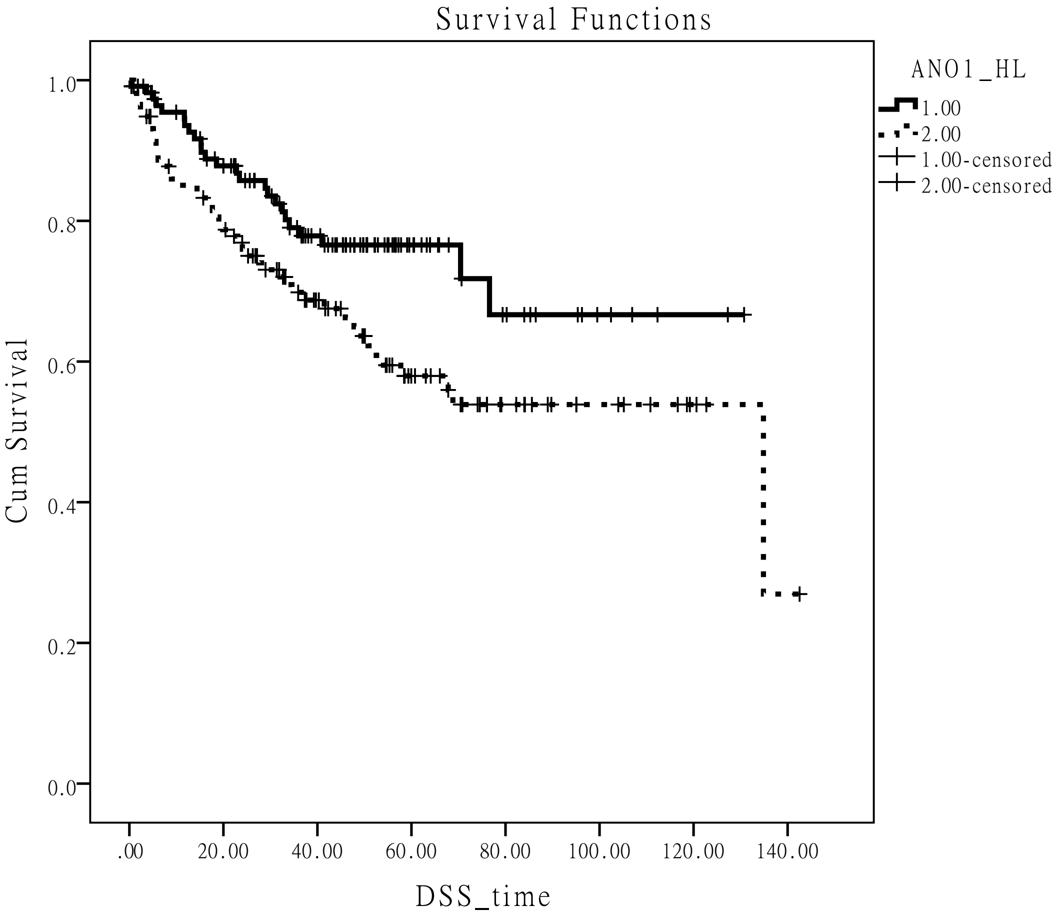


```
KM DSS_time BY SQLE_HL
  /STATUS=DSS(2)
  /PRINT MEAN
  /PLOT SURVIVAL
  /TEST LOGRANK BRESLOW TARONE
  /COMPARE OVERALL POOLED.
```

Kaplan-Meier

Notes

Output Created		07-Jun-2012 09:39:29
Comments		
Input	Data	C:\Users\hfyuen\Desktop\Axl-WWTR-CTGF-YAP1 in colon cancer\Threapeutic targets\GSE17536 and 537 combined_all hippo_coexpressed_therapeutics genes.sav
	Active Dataset	DataSet2
	Filter	<none>
	Weight	<none>
	Split File	<none>
	N of Rows in Working Data File	238
Missing Value Handling	Definition of Missing	User-defined missing values are treated as missing.
	Cases Used	Statistics are based on all cases with valid data for all variables in the analysis.
Syntax		KM DSS_time BY SQLE_HL<br>/STATUS=DSS(2)<br>/PRINT MEAN<br>/PLOT SURVIVAL<br>/TEST LOGRANK BRESLOW TARONE<br>/COMPARE OVERALL POOLED.
Resources	Processor Time	00 00:00:00.234
	Elapsed Time	00 00:00:00.258

[DataSet2] C:\Users\hfyuen\Desktop\Axl-WWTR-CTGF-YAP1 in colon cancer\Threapeutic targets\GSE17536 and 537 combined_all hippo_coexpressed_therapeutics genes.sav

Case Processing Summary

SQLE_HL	Total N	N of Events	Censored	
			N	Percent
1.00	114	25	89	78.1%
2.00	118	45	73	61.9%
Overall	232	70	162	69.8%

Means and Medians for Survival Time

SQLE_HL	Mean[a]				Median	
	Estimate	Std. Error	95% Confidence Interval		Estimate	Std. Error
			Lower Bound	Upper Bound		
1.00	101.932	5.028	92.077	111.788	.	.
2.00	86.987	6.116	75.000	98.973	134.860	40.236
Overall	95.656	4.597	86.647	104.665	134.860	41.419

Means and Medians for Survival Time

	Median	
	95% Confidence Interval	
SQLE_HL	Lower Bound	Upper Bound
1.00	.	.
2.00	55.998	213.722
Overall	53.678	216.042

a. Estimation is limited to the largest survival time if it is censored.

Overall Comparisons

	Chi-Square	df	Sig.
Log Rank (Mantel-Cox)	4.694	1	.030
Breslow (Generalized Wilcoxon)	4.009	1	.045
Tarone-Ware	4.281	1	.039

Test of equality of survival distributions for the different levels of SQLE_HL.

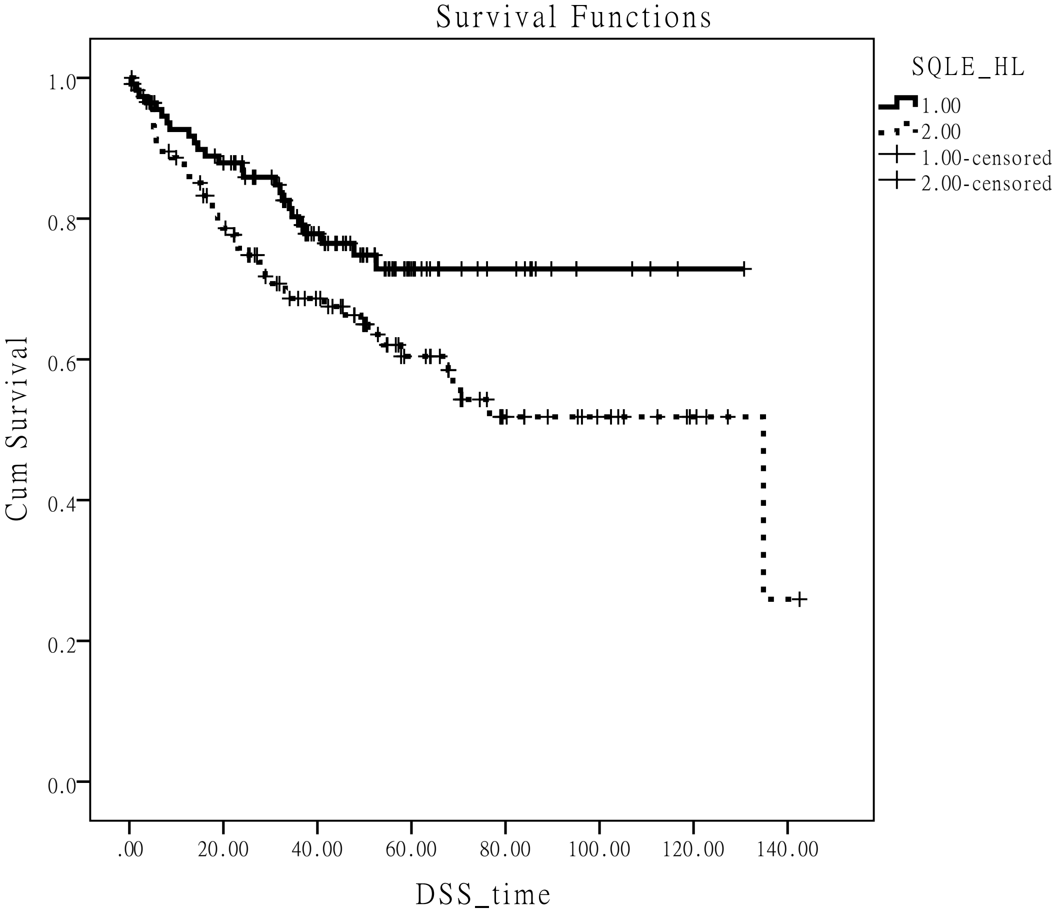

Supplement: S3 File — (ZIP) [file pone.0250187.s004.zip › Supplementary-Figure2-Kaplan-Meier-analyses-output.pdf]
